# Biogenic volatile release from permafrost thaw is determined by the soil microbial sink

**DOI:** 10.1038/s41467-018-05824-y

**Published:** 2018-08-24

**Authors:** Magnus Kramshøj, Christian N. Albers, Thomas Holst, Rupert Holzinger, Bo Elberling, Riikka Rinnan

**Affiliations:** 1 Terrestrial Ecology Section, Department of Biology, University of Copenhagen, Universitetsparken 15, DK-2100 Copenhagen, Denmark; 2 Center for Permafrost (CENPERM), Department of Geosciences and Natural Resource Management, University of Copenhagen, Øster Voldgade 10, DK-1350 Copenhagen, Denmark; 3 Department of Geochemistry, Geological Survey of Denmark and Greenland (GEUS), Øster Voldgade 10, DK-1350 Copenhagen, Denmark; 4 Department of Physical Geography & Ecosystem Science, Lund University, Sölvegatan 12, S-22362 Lund, Sweden; 5 Institute for Marine and Atmospheric Research (IMAU), Utrecht University, Princetonplein 5, 3584 CC Utrecht, The Netherlands

## Abstract

Warming in the Arctic accelerates thawing of permafrost-affected soils, which leads to a release of greenhouse gases to the atmosphere. We do not know whether permafrost thaw also releases non-methane volatile organic compounds that can contribute to both negative and positive radiative forcing on climate. Here we show using proton transfer reaction–time of flight–mass spectrometry that substantial amounts of ethanol and methanol and in total 316 organic ions were released from Greenlandic permafrost soils upon thaw in laboratory incubations. We demonstrate that the majority of this release is taken up in the active layer above. In an experiment using ^14^C-labeled ethanol and methanol, we demonstrate that these compounds are consumed by microorganisms. Our findings highlight that the thawing permafrost soils are not only a considerable source of volatile organic compounds but also that the active layer regulates their release into the atmosphere.

## Abstract (web summary)

Permafrost thaw allows biological activity in previously frozen ground, leading to a potential release of climate-relevant gases. Here the authors show that thawing permafrost emits volatile organic compounds but that their release to the atmosphere is limited by microbial consumption in the overlaying soil.

## Introduction

Aboveground vegetation is the most important source for biogenic volatile organic compounds (BVOCs) worldwide^1^. However, in the Arctic where plant biomass is low, the contribution of soils to ecosystem BVOC emissions is considered more important^2^. Soils release a large bouquet of compounds, with ethanol, methanol, acetone, acetaldehyde, and acetic acid belonging to the most commonly emitted chemical species^1,3^.

Almost half of the global below-ground carbon is stored in the Arctic permafrost soils and it is to a large extent unavailable for microbial degradation^4^. However, despite freezing temperatures in the permafrost, microbes remain live and active due to cold adaptations such as antifreeze proteins protecting the cell by lowering the freezing point of water^5^ and nucleic-acid-binding proteins relieving impaired access to RNA polymerase and increases in RNA secondary structures^6^. Permafrost hosts a large diversity of microbial life^7^, and habitats include liquid brine veins where the freezing point is depressed owing to high salt content, as well as the top permafrost infiltrated with melt water from the upper soil layers^7^. Permafrost soils have often been frozen for several thousand years, and in this period, products of the slowly occurring microbial activities buildup as greenhouse gases CO_2_, CH_4_, and N_2_O (refs. ^8,9^).

Owing to a number of climate system feedbacks, including albedo change due to retreating snow cover and sea ice, climate warming is particularly pronounced in the Arctic^10^. Consequently, the near-surface permafrost soils are projected to decrease by 37–81% by the year 2100 (ref. ^10^), and during this process large amounts of trace gases may be released to the atmosphere^11^. So far the release of BVOCs from thawing permafrost soil has not been studied, despite the fact that increased emission of these gases could have significant effects on the Arctic climate. BVOCs facilitate atmospheric particle growth, as their oxidation products condensate on aerosols^12^. This leads to both increased scattering of solar radiation and a higher concentration of cloud condensation nuclei, which in the unpolluted Arctic could significantly increase cloud formation and thus counteract the positive ice-albedo feedback^13^. Also, through a series of photochemical reactions, BVOCs can, in the presence of nitrous oxides, contribute to the formation of tropospheric ozone^14^, which is currently estimated to cause a 0.3 °C increase in surface temperatures^15^.

In three separate laboratory experiments, we examine the release of BVOCs from thawing permafrost soils and follow the fate of these compounds in the active layer soil. First, we incubate frozen permafrost soils in a dynamic flow-through system and use a proton transfer reaction–time of flight–mass spectrometer (PTR-TOF-MS) instrument to detect 316 organic ions being released. To assess whether these compounds are likely to end up in the atmosphere or be retained in the active layer soil overlaying the permafrost, we perform a second experiment, estimating the BVOC uptake capability of organic and mineral horizon soils. The results show that the majority of BVOCs released from the permafrost were retained in the active layer soil. We therefore go on to investigate whether microbial consumption of the two dominating BVOCs, ethanol and methanol, occurred in the studied active layer soils as well as the permafrost soil at low temperatures and realistic mixing ratios. We find that both ethanol and methanol were rapidly consumed by microbes. Our results show that thawing permafrost releases significant amounts of BVOCs and that these compounds are consumed by microbes in soil from the active layer.

## Results

### Release of volatiles from permafrost during thaw

We incubated frozen permafrost soils in a dynamic flow-through system and measured BVOC release during and after thaw at a temperature of 6 °C for 43 h using a PTR-TOF-MS instrument (from hereafter referred to as the Release experiment). The permafrost soils were sampled along a south-facing tundra slope on Disko Island, Western Greenland (69°16’N, 53°27’W) in August 2015. We calculated emission rates for a total of 316 different organic ions, with mass-to-charge ratios (*m*/*z*) between 26 and 280. Ethanol (detected at *m*/*z* 47.049) and methanol (*m*/*z* 33.033) were the most abundant compounds released from the permafrost, accounting for 51% and 26% of the total release, respectively. Acetaldehyde (*m*/*z* 45.033), acetone (*m*/*z* 59.049), and formaldehyde (*m*/*z* 31.018) accounted for 7.5%, 5.1%, and 3.9% of the release, respectively (Table 1, Supplementary Fig. 1). A complete list of released ions is shown in Supplementary Data 1.

**Table 1:** The most emitted compounds

Compound	Mass-to-charge ratio	Emission rate (nmol g^−1^ dw soil h^−1^)	Relative abundance (%)
Ethanol	47.049	1.345	51.2
Methanol	33.033	0.673	25.6
Acetaldehyde	45.033	0.198	7.5
Acetone	59.049	0.134	5.1
Formaldehyde	31.018	0.103	3.9
Acetonitrile	42.034	0.062	2.4
2-Butanone	73.064	0.023	0.9
2-Butene/2-methyl-1-propene	57.069	0.010	0.4
Propyne/1.2-propadiene/cyclopropene	41.038	0.009	0.3
Cyclopropane/propene	43.054	0.008	0.3

Emission rate and relative abundance of the ten compounds released from permafrost soils in highest quantities is presented

*dw* dry weight

**Fig. 1:**
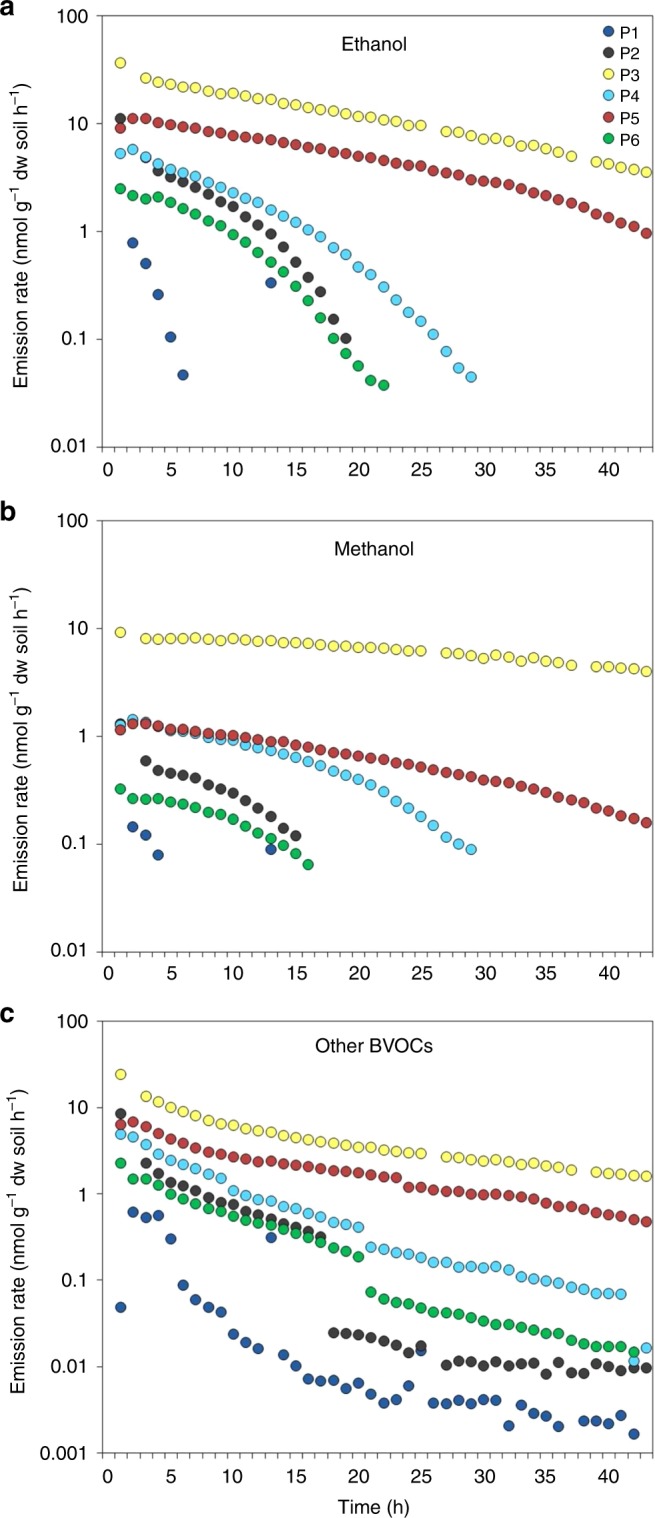
Release of volatiles from thawing permafrost soils. The release of **a** ethanol, **b** methanol, and **c** other biogenic volatile organic compounds (BVOCs) over 43 h is shown for six permafrost soils (P1–P6) collected on Disko Island, Greenland. dw dry weight

The rate of total BVOC emission peaked within the first hours for all permafrost soils and ranged from 0.8 to 63.8 nmol g^−1^ dry weight soil h^−1^. The emission rate decreased considerably during the incubation for all six soils. After 43 h, four soils released <0.02 nmol g^−1^ dw soil h^−1^, while two soils had emission rates of 1.6 and 9.0 nmol g^−1^ dw soil h^−1^, following a continuously declining trend (Fig. 1).

**Fig. 2:**
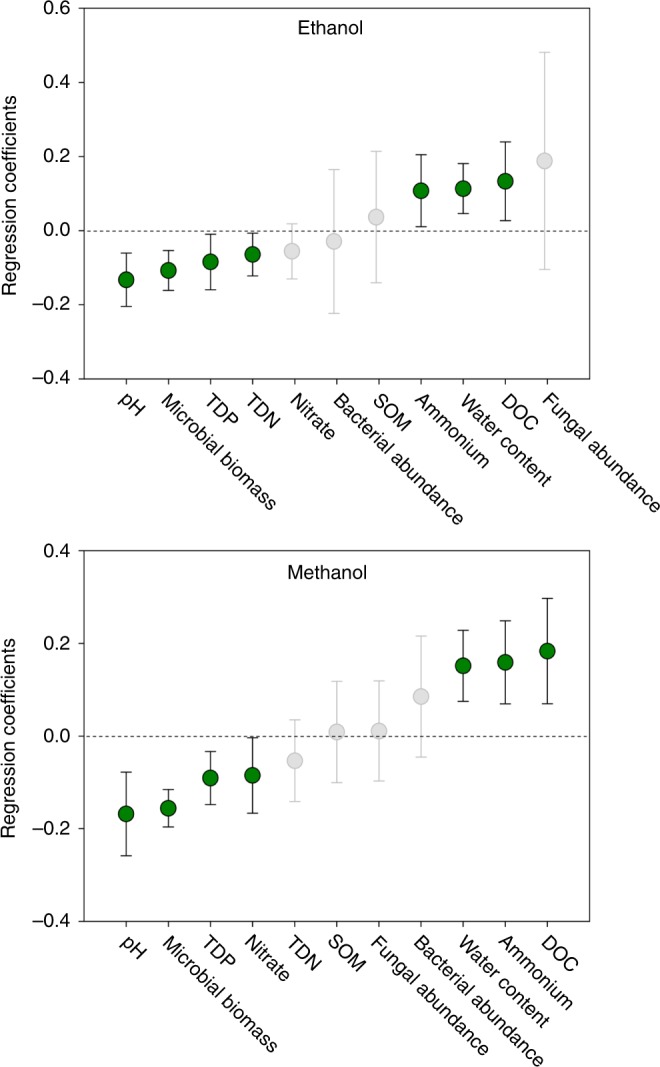
Correlation between permafrost soil characteristics and BVOC release during permafrost thaw. Regression coefficients of partial least squares regression (PLSR) models for the covariance between the measured soil variables and the accumulated release of **a** ethanol and **b** methanol during the first 5 h of permafrost thaw. All models had one PLS component. Positive regression coefficients indicate a positive relationship and negative ones a negative relationship. Error bars show ± confidence intervals (95%) of the regression coefficients. Significant factors are shown in green

The organic matter content and the gravimetric water content of the permafrost samples averaged 28% and 48%, respectively, with considerable variation between individual samples (Table 2). Owing to the high water content, the permafrost samples have most likely contained anaerobic microsites during the experiments. To examine whether the measured soil characteristics (Table 2) could explain variation in emission rates of the ten most emitted compounds between the permafrost samples, we conducted partial least squares regression (PLSR) analyses. We found that the emissions of ethanol, methanol, and most other compounds positively correlated with the dissolved organic carbon (DOC) and ammonium concentrations as well as the water content of permafrost (Fig. 2, Supplementary Fig. 2, 3). The variables showing strongest negative correlations with the emission rates were pH, microbial biomass, and water-extractable dissolved phosphorus concentration. Also total dissolved nitrogen and nitrate concentrations correlated negatively with the emission rates, although this was not significant for all BVOCs. For acetonitrile, bacterial abundance showed significant positive correlation (Supplementary Fig. 2).

**Table 2:** Soil characteristics

	P1R	P2R	P3R	P4R	P5R	P6R	P1U	P2U	P3U	P4U	P5U	P6U
pH	6.9	6.3	5.9	6.2	6.1	6.9	6.9	6.3	5.9	6.5	6.2	6.2
Soil organic matter (%)	11.2	34.4	43.2	26.7	25.7	55.6	37.9	37.0	15.6	11.8	11.7	24.0
Gravimetric water content (%)	37	62	72	44	48	26	32	34	59	61	62	44
DOC (μg g^−^^1^ dw soil)	65	170	268	138	143	105	65	170	268	128	226	72
NO_3_-N (ng g^−^^1^ dw soil)	389	526	117	31	24	98	389	526	117	83	96	73
NH_4_-N (ng g^−1^ dw soil)	63	137	599	236	145	—	63	137	599	520	593	134
TDN (μg N g^−^^1^ dw soil)	19.3	16.0	14.9	15.2	11.0	18.5	19.3	16.0	14.9	19.0	11.4	8.0
TDP (μg PO_4_ g^−^^1^ dw soil)	1.1	0.4	0.2	0.2	0.2	3.8	1.1	0.4	0.2	0.4	0.2	0.2
Bacterial abundance (10^9^ × 16S gene copies g^−1^ fw soil)							2.3	3.0	6.7	3.9	6.3	9.4
Fungal abundance (10^5^ × ITS2 gene copies g^−1^ fw soil)							5.7	0.4	2.9	1.6	2.1	2.8
Microbial biomass (μg g^−1^ dw soil)	367	232	203	291	317	352	367	232	203	303	128	319

Soil parameters measured in permafrost soil core samples. Gravimetric water content, soil organic matter, bacterial abundance, and fungal abundance were measured on soil samples incubated in the experiments, while other parameters were measured on homogenized bulk soil

P1–P9, individual permafrost soil samples; R, Release experiment; U, Uptake experiment; DOC, dissolved organic carbon; TDN, total dissolved nitrogen; TDP, total dissolved phosphoru;s fw, fresh weight; dw, dry weight

### Uptake of volatiles from permafrost by active layer soil

**Fig. 3:**
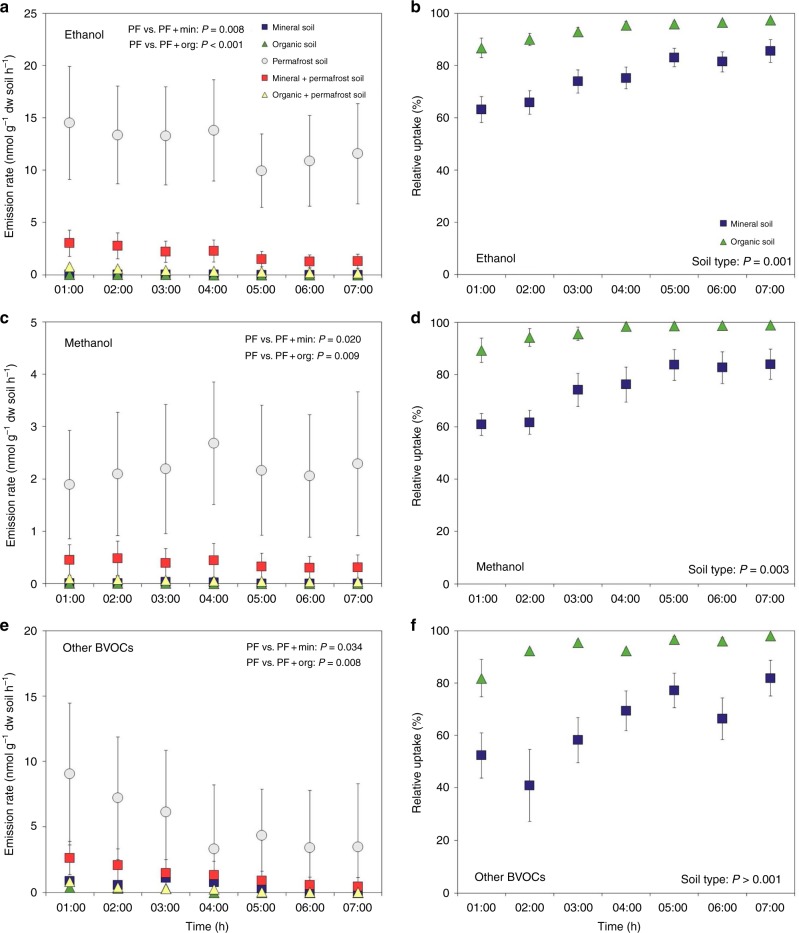
Uptake of volatiles by mineral and organic active layer soils. Mean release rate (*n* = 6) is shown for **a** ethanol, **c** methanol, and **e** other biogenic volatile organic compounds (BVOCs) from incubations of mineral soil, organic soil, permafrost soil, permafrost+mineral soil, and permafrost+organic soil. The relative uptake by mineral and organic active layer soils of **b** ethanol, **d** methanol, and **f** other BVOCs released by permafrost soils is furthermore presented. Statistically significant *P* values for the univariate analyses of variance (ANOVA) are shown. In **a**, **c**, **e**, *P* values from Dunnett’s test represent the difference in BVOC release between permafrost soil (PF) and permafrost+mineral soil (PF+Min) or permafrost+organic soil (PF+Org). In **b**, **d**, **f**, *P* values represent the effect of soil type on BVOC uptake. Error bars show standard error of the mean

To assess whether the BVOCs released from thawing permafrost are likely to end up in the atmosphere or be retained in the active layer soil overlaying the permafrost, we performed an additional uptake experiment (from hereafter referred to as the Uptake experiment). BVOC release was measured for the permafrost soils alone as well as together with either the organic or mineral horizon of the active layer soil sharing the incubation atmosphere but without physical contact between the two soils. After 1 h at 6 °C, the first measurements showed that organic and mineral active layer soils took up 85% and 60% of the BVOCs released from the permafrost (See Fig. 3 for ethanol, methanol, and total other BVOCs and Supplementary Figs 4–6 for the most abundant other compounds). After 7 h, the relative uptake had increased to 98% and 86%, respectively.

### Mineralization of ethanol and methanol by soil microbes

We went on to investigate whether microbial consumption of ethanol and methanol occurs in the studied mineral and organic active layer soil as well as the permafrost soil at low temperatures and realistic mixing ratios (from hereafter referred to as the Mineralization experiment). We injected ^14^C-labeled ethanol (2.3−3.6 ng g^−1^ fresh weight soil) and methanol (1.1–1.8 ng g^−1^ fresh weight soil) to the headspace of incubators with soil samples and followed over time the mineralization of the compounds to CO_2_ at 6 °C.

**Fig. 4:**
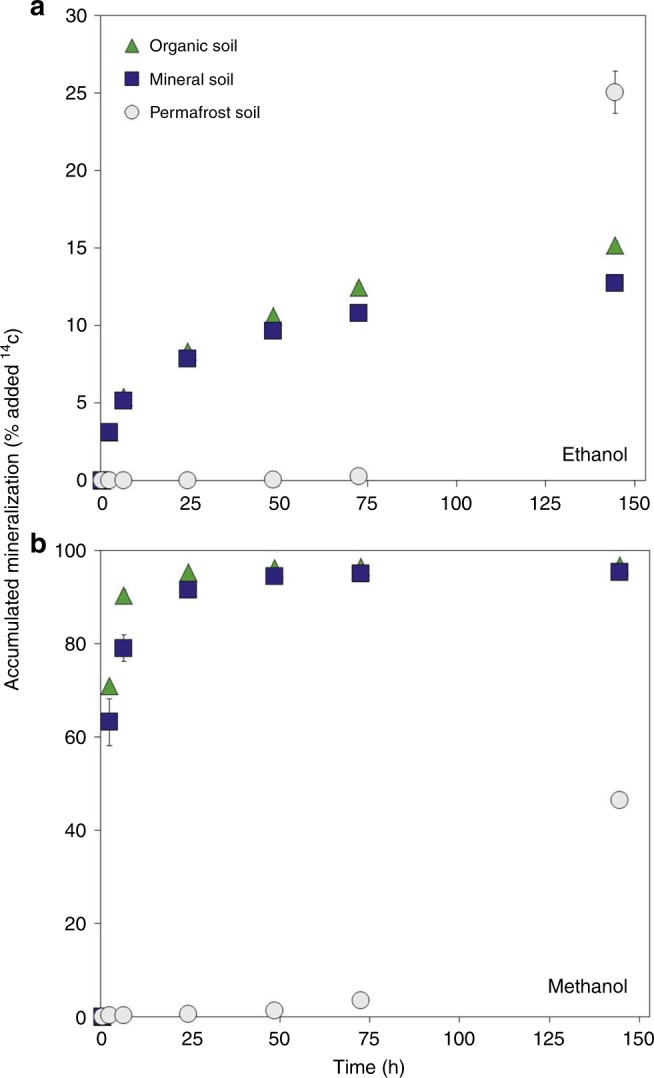
Microbial mineralization of ethanol and methanol. Mean mineralization rates (*n* = 3) are shown for ^14^C-labeled **a** ethanol and **b** methanol in organic, mineral, and permafrost soils. Initial concentrations were approximately 3 and 1.5 µg kg^−^^1^ fresh weight soil for ethanol and methanol, respectively. The incubation temperature was 6 °C. Sterilized soil working as negative controls showed no mineralization. Error bars show standard error of the mean. Some error bars are smaller than the symbols

The mineralization to CO_2_ occurred faster and to a much higher extent for methanol compared to ethanol (Fig. 4). After 2 h, 3% of the added ethanol was mineralized to CO_2_ in both organic and mineral soil layers, while 70% and 60% of the added methanol was mineralized in organic and mineral soils, respectively. Ultimately, about 95% of the added ^14^C-methanol was transformed into ^14^C-CO_2_, while that was the case for only 15% of ethanol (Fig. 4). However, for both compounds, most of the mineralization occurred within the first 24 h followed by a low linear increase for the remaining incubation period. No ^14^C could be extracted with an organic solvent from the soils at the end of the experiment, suggesting that all the added ^14^C-labeled ethanol and methanol was either converted into ^14^C-CO_2_ or incorporated into the microorganisms.

During the first 72 h, there was, in contrast to the active layer soils, hardly any mineralization of ethanol or methanol in the permafrost soils that had been thawed and stored at 4 °C 1 day prior to the experiment. Between 72 and 144 h, mineralization activity increased, and 45% of the ^14^C-methanol and 25% of the ^14^C-ethanol was mineralized.

To test whether the observed mineralization could be due to abiotic degradation of ethanol and methanol, we included soil samples sterilized by autoclaving in the experiment. In the sterilized samples, only 1% of the methanol and none of the ethanol was mineralized to CO_2_ after 144 h.

## Discussion

We have demonstrated that permafrost soils upon thaw release a variety of BVOCs with ethanol and methanol as the dominant compounds. The release rate of BVOCs peaked within the first 2 h of thaw and then declined over time. This rapid release and the subsequent decline in BVOC release has two potential explanations: previous production processes may have occurred in the frozen permafrost soil^16–18^, slowly causing a buildup of immobilized BVOCs that were now released. Alternatively, the turnover of a limited pool of labile carbon made available upon thaw^19^ could cause an initially high microbial fermentation rate and a release of trace gases that decreases over time^20^. In Arctic active layer soils, a large ethanol production has been shown to correlate with a depletion of labile carbon made available upon thaw^20^. However, the rapid appearance of the release peak at low temperature (6 °C) points to a release of previously trapped gases rather than post-thaw microbial production, as earlier shown for methane^17,21^.

Ethanol is produced from fermentative degradation of plant residuals in anoxic soils by microorganisms, facilitated by the enzymes pyruvic decarboxylase and alcohol dehydrogenase^22^, while methanol can be formed by chemical and enzymatic demethylation of the methoxy groups of pectin in decaying plant cell walls^23^. Furthermore, compounds such as ethanol, methanol, and acetone can be produced in non-enzymatic thermochemical Maillard reactions^24^. Such reactions are, however, strongly temperature-dependent, and owing to the low incubation temperature used in our experiments, most likely only a minor part of the observed BVOC release is derived from abiotic processes.

DOC concentration, ammonium concentration, and water content of the permafrost were the soil properties correlating strongest with BVOC emissions during permafrost thaw, suggesting that the most fertile soils with potentially highest degree of anoxia due to waterlogging had the highest net production potential for the studied BVOCs. Microbial biomass and pH show a negative correlation with BVOC release, which can be due to an inverse relationship with water content of the soil as soils with a higher water content and therefore more anoxic conditions had lower pH and lower microbial biomass.

Despite the significant release of BVOCs from the thawing permafrost soils, it is likely that most of these compounds will never reach the atmosphere. In the Uptake experiment, both the mineral and organic horizon soil from the active layer took up the majority of the BVOCs released from the thawing permafrost. This uptake could be caused by BVOC sorption to soil particles and organic material, dissolution in the water phase for hydrophilic compounds, or microbial consumption. All three processes occur to some extent, but our PTR-TOF data suggest that microbial uptake was the major removal process. We observed that the relative BVOC uptake increased with time and that owes support to biotic rather than physico-chemical processes. In physico-chemical processes, the relative uptake would be expected to decrease rather than increase, as adsorption or dissolution would likely be reversible in contrast to microbial degradation of the compounds.

In the Mineralization experiment, we showed that added ^14^C-ethanol and ^14^C-methanol is rapidly converted to ^14^CO_2_. This conversion did not take place in sterilized soil samples indicating that it was in fact a result of microbial activity. Degradation of both ethanol and methanol started immediately in the active layer soils. The mineralization to CO_2_ occurred faster and to a higher extent for methanol compared to ethanol (Fig. 4). However, at the end of the experiment, no ^14^C could be extracted from the soils, suggesting that also all of the added ethanol was quickly degraded microbially, just with a lower yield as ^14^CO_2_. A wide range of soil microorganisms utilize ethanol and methanol as a carbon and energy source under both oxic and anoxic conditions^22,25,26^. The fact that these compounds were mineralized by microbes in the two active layer soils is therefore not in itself surprising, but the fact that the microbes do degrade the low concentrations at a very high pace and with different ^14^CO_2_ yields is interesting. Both ethanol and methanol were degraded very fast (complete within 24 h and in the case of methanol probably much faster) but with different utilization strategies. Methanol seems to be used only as energy source (high ^14^CO_2 _yield), while ethanol is used mainly as a source of carbon for microbial growth and synthesis of molecules that are then slowly mineralized to CO_2_ (low ^14^CO_2 _yield). This is in agreement with the known degradation pathways of the two compounds. Methanol degrades through formaldehyde and formate to CO_2_, while ethanol is degraded through acetaldehyde to acetyl-CoA, which can be used as a building block for fatty acids, amino acids, etc.^27^.

As opposed to the active layer soils, degradation of ethanol and methanol in permafrost did not start until 72–144 h of incubation. This is in agreement with previous experiments with permafrost soil showing that microbial respiration starts to increase 3 days after thaw and peaks after 2 weeks^28^. The initial lag phase can be explained by the fact that microorganisms may need some time to adjust to the new conditions and become active or that microbial growth was needed to obtain the increase in activity^21,28^.

The fast and complete degradation of ethanol and methanol to CO_2_ in the active layer soils proves that the BVOC sink is primarily due to microbial mineralization rather than chemical or physical retention. In accordance with previous studies^29^, we found higher microbial abundance in organic than in mineral horizon of the active layer soil (Table 2), which could explain the slightly higher capability of organic soils to take up BVOCs (Figs. 3, 4). The permafrost samples had a very low abundance of fungi but only ten times less bacteria compared to the organic soil (Table 2). The much lower degradation of BVOCs in the permafrost as observed in both the Uptake and Mineralization experiments thus shows that either the bacterial community in the permafrost is not yet suited for BVOC degradation or that it is simply not as active as that in the active layer soils.

The importance of the microbial uptake is likely dependent on the ice content of the thawed permafrost and contrasting drainage conditions of the active layer across the landscape, both factors determining the water and oxygen content of the soil. In waterlogged soils, microbial BVOC mineralization rate is probably low, and a larger part of the BVOCs released from thawing permafrost could thus reach the atmosphere.

As the maximum emission rates observed suggest that permafrost may be a significant source of BVOCs to the Arctic atmosphere, it is important to further address the processes of releasing and consuming BVOCs in soil in future studies. To provide a more precise estimate of the future Arctic soil BVOC emissions under permafrost thaw, studies covering different permafrost types and focusing on different drainage conditions are needed. Furthermore, permafrost soil can be exposed and come directly in contact with the atmosphere, for instance, along shores and rivers^11^. Also, owing to the annual freezing and thawing, Arctic soils are dynamic and in some cases frost heaving can lift up permafrost layers so that BVOCs would bypass the active layer and be released directly to the atmosphere upon thaw.

## Methods

### Site description

Permafrost cores and active layer soil samples were collected in early August 2015 in the Blæsedalen Valley, located on Disko Island in West Greenland (69°16’18.03”N; 53°28’11.93”W). The climate is Low Arctic and a climate station located near the sea level reveals a mean annual soil temperature at 5 cm depth of 0.9 °C (1991–2004) and an annual mean air temperature of −3.0 °C (1992–2012)^30,31^. The geomorphology is formed by the Weichselian ice age and the deglaciation during the past 10,000 years. Main features are glacial valleys eroded into basaltic layers and deposited tills. The sampling site is situated on a south-facing slope about 110 m a.s.l. on the foot of a moraine with an average maximum depth to the permafrost table between 40 and 60 cm. An active layer is developing each year from late May until October and the top of the soil profile is well drained and the soil development weak but with a distinct A-horizon of up to 20 cm. The vegetation in the area is dominated by *Empetrum nigrum*.

The site was selected as being as representative as possible for slopes in West Greenland. Located at the border between High and Low Arctic, the site is expected to be in the front line of climate change and the change from continuous to discontinuous permafrost.

### Permafrost and active layer sampling

Nine independent intact permafrost samples were sampled from pits by the use of sterilized steel pipes hammered into the permafrost ca. 10 cm below the permafrost table. The permafrost soils were kept frozen during transport to Copenhagen. In Copenhagen, the soil cores were homogenized into particles <1 cm^3^ in a freezer room using a hammer and a metal mesh (8 mm mesh size). Permafrost soils used in the Release and Uptake experiments were stored at −6 °C for 6 months, transported to the analysis laboratory, and stored there at −18 °C for 10–15 days prior to the start of the experiment. Permafrost soils used in the Mineralization experiment were stored at −6 °C and thawed at 4 °C 1 day before the experiment.

Soil cores of the active layer were sampled by pushing a brass tube (diameter 4 cm) 10 cm into the soil after carefully removing the vegetation. The organic soil horizon (depth varied between 3 and 7 cm) was then separated from the mineral horizon. The active layer soils were stored at −6 °C and then thawed at 4 °C approximately 10 days prior to the start of each experiment. Shortly after the soils had thawed, they were gently homogenized by hand wearing gloves, followed by removal of roots and stones >3 mm in diameter.

### Soil characterization

The frozen soil samples were thawed at 5 °C for 24 h prior to the chemical and microbial analysis. The samples were analyzed in duplicates as described below. Gravimetric soil water content was determined based on the water loss after drying at 70 °C for 24 h. Soil organic matter content was determined by loss on ignition at 550 °C for 6 h.

For microbial biomass estimation, 5 or 10 g of fresh soil (depending on the soil sample) was fumigated with ethanol-free chloroform for 24 h to lyse microbes. After fumigation, the soil samples were extracted in 25 ml (for 5 g soil samples) or 50 ml (for 10 g soil samples) deionized water on a rotary shaker for 1 h (ref. ^32^). A second, non-fumigated subsample of each sample was extracted in a similar way. All extracts were filtered through GF-D glass microfiber filters (Whatman Ltd., Maidstone, UK) and stored at 5 °C until analysis.

DOC in the extracts was analyzed with a TOC-L total organic carbon analyzer (Shimadzu, Kyoto, Japan). Inorganic nitrogen (NH_4_^+^-N and NO_3_^−^-N), total dissolved N (TDN) and total dissolved phosphorus (TDP) were measured using a FIA STAR 5000 flow injection analyzer (FOSS Teactor, Höganäs, Sweden). Before TDN and TDP measurements, the extracts were digested in persulfate and selenic acid, respectively. Blank samples only containing deionized water were analyzed as a reference. Microbial biomass C was calculated as the difference in DOC between non-fumigated and fumigated extracts, and a conversion value (*k*_EC_) of 0.45 was used to compensate for incomplete extractability^32^.

For all soil types, triplicated 0.25 g DNA extraction subsamples were made using the PowerLyzer PowerSoil DNA Isolation Kit (MoBio, Carlsbad, California). Total fungal biomass was quantified based on the number of ITS2 gene copies obtained by targeting the fungal ITS2 nuclear ribosomal DNA region using forward primer gITS7 (GTGARTCATCGARTCTTTG) and reverse primer ITS4 (TCCTCCGCTTATTGATATGC) as described by Christiansen et al.^33^. Total bacterial biomass was quantified based on the number of 16s gene copies obtained by quantitative PCR targeting the 16S rRNA sequence using forward primer 341 F (5′-CCTACGGGAGGCAGCAG-3′) and reverse primer 518 R (5′-ATTACCGCGGCTGCTGG-3′) and 1 µl DNA template, as previously described by Feld et al.^34^.

Soil characteristics in the active layer soils are presented in Supplementary Table 1.

### BVOC release from thawing permafrost

For the BVOC measurements in the Release experiment, six 13-g fresh weight frozen permafrost soil samples were placed in polyester woven mesh bags (mesh size 515 micron) and incubated in 200 ml glass jars sealed by aluminium covered screw lids at 6 °C for 43 h. The glass jars, lids, and mesh bags had been carefully cleaned and heated at 120 °C for 2 h prior to all measurements.

Outdoor air was pushed through a zero-air generator (Parker Balston, Haverhill, USA) and a flow of 400 ml min^−1^ was equally diverted into two PFA lines (Swagelok, Solon, USA) feeding 3 sample glass jars each. A Teflon solenoid valve (Parker Hannifin, Cleveland, USA) opened one of the three sample jars in each PFA line based on a datalogger-controlled (Campbell Sci, Logan, USA) schedule, and a three-way Teflon solenoid valve (Parker Hannifin, Cleveland, USA) was alternating between these two excess lines every 10 min to let the PTR-TOF-MS draw a subsample of 100 ml min^−1^ for BVOC analysis. Flows entering and leaving the sample jars were kept constant and monitored using flow meters (Alicat Scientific, Tucson, USA and Key Instruments, Trevose, USA).

The experimental set-up included an empty jar that contained no soil but similar mesh bags as with the soil samples. The empty jar was measured every hour and served as blank sample to be able to account for potential VOC contamination in the used zero air or release of compounds from the used materials.

All sample jars and valves to open and close the lines were kept at the same temperature as the soil (6 °C), but lines that were used to transfer sample air to the PTR-TOF-MS were kept at room temperature (ca. 22 °C) before entering the PEEK capillary inlet system of the PTR-TOF-MS operating at 60 °C. Thus temperatures from the sample jars were increasing toward the instruments, preventing condensation in the lines.

With this set-up, all 6 jars were measured for BVOC release for 10 min followed by flushing for 10 min once per hour. Equilibrium was reached after a few minutes (Supplementary Fig. 7), and the emission rate was calculated based on the average incubation concentration after 4–8 min of sampling.

### BVOC removal by active layer soil

The Uptake experiment consisted of incubations of five different sample set-ups: organic soil alone, mineral soil alone, permafrost soil alone, organic soil plus permafrost soil, and mineral soil plus permafrost soil (Supplementary Fig. 8). In addition to these five set-ups, an empty glass jar with mesh bags was measured throughout the experiment. The soil samples weighed 13 g fresh weight each and the incubations with two soil types thus contained 26 g fresh weight soil in total.

Incubations of the organic and mineral soils were conducted as for the permafrost samples except for the incubations that contained two soil types. Here each soil type was placed in a separate mesh bag with the active layer soil on top of the permafrost soil, the mesh bag ensuring no physical contact between the soils. The emissions from the incubators were sampled for 7 h and the technical set-up was as described for the Release experiment.

### BVOC analysis instrumentation

BVOC mixing ratios were measured using PTR-TOF-MS (PTR-TOF-MS 8000, Ionicon Analytik, Innsbruck, Austria). The PTR-TOF-MS was operated at 60 °C drift tube temperature, 2.20 hPa drift tube pressure, and 550 V drift tube voltage, which led to an *E* over *N* ratio (electric field *E*, number density *N*) of ca. 130 × 10^−21^ V m^2^ = 130 Td. Compounds up to *m/z* = 280 Da were monitored at 1 Hz temporal resolution. The instrument provides a mass resolution >4000 m/∆m (full width half maximum) and a detection limit <10 ppt (ref. ^35^). The instrument was equipped with a heated PEEK capillary inlet system (operated at 60 °C) and a built-in permeation unit (PerMaSCal; Ionicon Analytik, Innsbruck, Austria) which emitted 1,3-diiodobenzene (C6H4I2), which was used for an improved mass scale calibration by adding a continuous strong signal at a higher mass peak (i.e., *m*/*z* 203.943). Details about the PTR-TOF-MS technique are available from Lindinger et al.^36^ and Graus et al.^35^.

### Data processing

Data from the PTR-TOF-MS was processed using the PTRwid software tool^37^. PTRwid was used to detect and identify mass peaks in the measurement spectrum, to calibrate the mass scale automatically, to convert the count rates of the detected compounds to VOC mixing ratios (following Holzinger et al.^38^), and finally to provide output files with time-averaged (10 s) VOC mixing ratios of all detected mass peaks. VOC concentrations in blank samples were subtracted from those in the soil samples. The concentrations in the blanks were in general very low, suggesting minimal or no contamination from the zero air or used materials.

### Chemical composition determination

The molecular formula was identified (ion mass range within ±3 mDa) with a combination of carbon, hydrogen, oxygen, and nitrogen atoms using the database created by Holzinger et al.^37^, comprising ~18,000 possible molecular formulas. A possible molecular formula was not identified for ions above *m*/*z* 237, as they tend to adsorb to instrumental parts and concentration estimation therefore is highly uncertain^39^. In the calculation of total BVOC carbon emission for the compounds for which no molecular formula had been identified, an average of all the possible formulas was used. Complete lists of released ions from organic, mineral, and permafrost layer soils are given in Supplementary Data 1.

### Calibration

While the PTRwid software already applied a mass-dependent transmission efficiency of the detector (based on Cappellin et al.^40^) and a default reaction rate coefficient for the protonation in the drift tube when converting from count rate to mixing ratio, the accuracy can be improved when calibrating the instrument against a gas standard. The PTR-TOF-MS was calibrated against a gas standard mixture (ca. 1 ppmv, Ionicon Analytik, Innsbruck, Austria) at different concentrations by diluting the gas mixture with zero air (GCU-b, Ionicon Analytik, Innsbruck, Austria) before, during, and after the experiments. Compound-specific calibration coefficients were averaged and applied for the whole period of the experiment for a number of compounds (Supplementary Table 2). The accuracy for estimation of the concentrations of the compounds without specific calibration standards was estimated to be ±40% for the ions with *m*/*z* <150 and ±60% for the ions with *m*/*z* above.

### BVOC mineralization experiment

The mineralization rates of ethanol and methanol to CO_2_ were tested in organic, mineral, and permafrost layer soils using a recently developed method based on ^14^C-labeling (ref. ^41^). Briefly, 6 g fresh weight (organic horizon) or 10 g (mineral horizon and permafrost) soil samples were weighed into 120 ml serum flasks in triplicates and incubated at 6 °C. In each flask, a glass vial containing 2.5 ml 1 M NaOH and 0.01 M NaHCO_3_ was placed to trap ^14^CO_2_ mineralized from ^14^C-labeled ethanol/methanol.

Stock solutions (3 × 10^7^ DPM ml^−1^) of ^14^C-labeled ethanol (55 mCi mmol^−1^) and methanol (58 mCi mmol^−1^) were made in sterile water and 0.5 ml was then distributed across the soil with a pipette. The ethanol and methanol concentrations in the incubations were about 3 nmol l^−1^ (1–4 µg kg^−1^) and probably slightly biased as a fraction of the ethanol/methanol was dissolved in water or adsorbed to the soil.

The flasks were closed with crimp-caps containing an alumina-coated septum (Mikrolab Aarhus, Denmark) and incubated at 6 °C following addition of ethanol/methanol. The CO_2_-trap was exchanged through a needle syringe permanently installed in the septum at six time points during the experiment. To be able to differentiate between the trapped ^14^CO_2_ and the dissolved ^14^C-labeled ethanol/methanol, the CO_2_-trap was split in two. One ml of the CO_2_-trap was transferred to a 2 ml Eppendorf tube with 0.7 ml water and the other 1 ml of the CO_2_-trap was transferred to a 2 ml Eppendorf tube with 0.7 ml BaCl_2_ (1.7 M) to precipitate the trapped ^14^CO_2_ as Ba^14^CO_3_. The samples were then left for 5 h to react, followed by 2 min centrifugation at 12,000 × *g*, and then 1 ml from each tube was mixed with HiSafe 3 LSC-cocktail (Perkin Elmer, Waltham, MA) and counted for 30 min by liquid scintillation (Tri-Carb 2810 TR, PerkinElmer, Waltham, MA).

The last sample was taken after 144 h, and then 30 ml methanol was added to each flask through the permanently installed needle to extract any residual ^14^C-ethanol or -methanol. The samples were shaken for 24 h, and the supernatant was transferred to a 50 ml centrifuge tube and centrifuged for 10 min at 6000 × *g*. The amount of extractable ^14^C (non-degraded ethanol and methanol) was then determined in 3 ml supernatant by liquid scintillation counting.

Negative controls of each soil type were included in which the soil had been sterilized by autoclaving twice.

### Statistical analyses

PLSR in SIMCA 13.0.3 (Umetrics, Umeå, Sweden) was used to assess for covariance between the permafrost soil characteristics and the emission rates of the ten most emitted BVOCs. PLSR is a flexible multivariate technique used to predict a *Y* variable (here BVOC emission rate) with a number of *X* variables (here the soil characteristics presented in Table 2), which can be correlated. The data were centered and all variables were auto-scaled to unit variance to give equal importance for all variables. The one-component PLS models were cross-validated using six cross-validation groups.

For the Uptake experiment, the difference in BVOC release for permafrost alone as compared with permafrost soil together with organic or mineral soil was statistically tested in IBM SPSS Statistics (Version 22.0, IBM Corp., New York City, United States). The mean difference in BVOC release over time between incubations with and without active layer soil was tested in General Linear Model Univariate Analysis of Variance (ANOVA) with soil type (permafrost alone; organic plus permafrost soil; mineral plus permafrost soil) as a fixed factor and mean BVOC release over time as a dependent variable. Dunnett post hoc test was used for comparisons of the permafrost alone against permafrost with organic and mineral soils. The differences in the relative BVOC uptake between organic and mineral layer soils were tested with General Linear Model Repeated Measures ANOVA with time as a within-subjects factor and active layer type (organic or mineral) as a between-subjects factor.

## Electronic supplementary material

Supplementary Information

Peer Review

Description of Additional Supplementary Files

Supplementary Data 1

## Data Availability

The authors declare that the data supporting this study are available within the paper and its Supplementary Information. All data are also available from the authors upon request.
