# Supplementary material for: Biogenic volatile release from permafrost thaw is determined by the soil microbial sink
Source: Nat Commun. 2018 Aug 24;9:3412. doi: 10.1038/s41467-018-05824-y (PMC6109083; doi:10.1038/s41467-018-05824-y)
Supplement: Supplementary file 1 — Supplementary Information [file 41467_2018_5824_MOESM1_ESM.pdf]

Supplementary Information for:

Biogenic volatile release from permafrost thaw is determined by the soil microbial sink

Kramshøj et al.

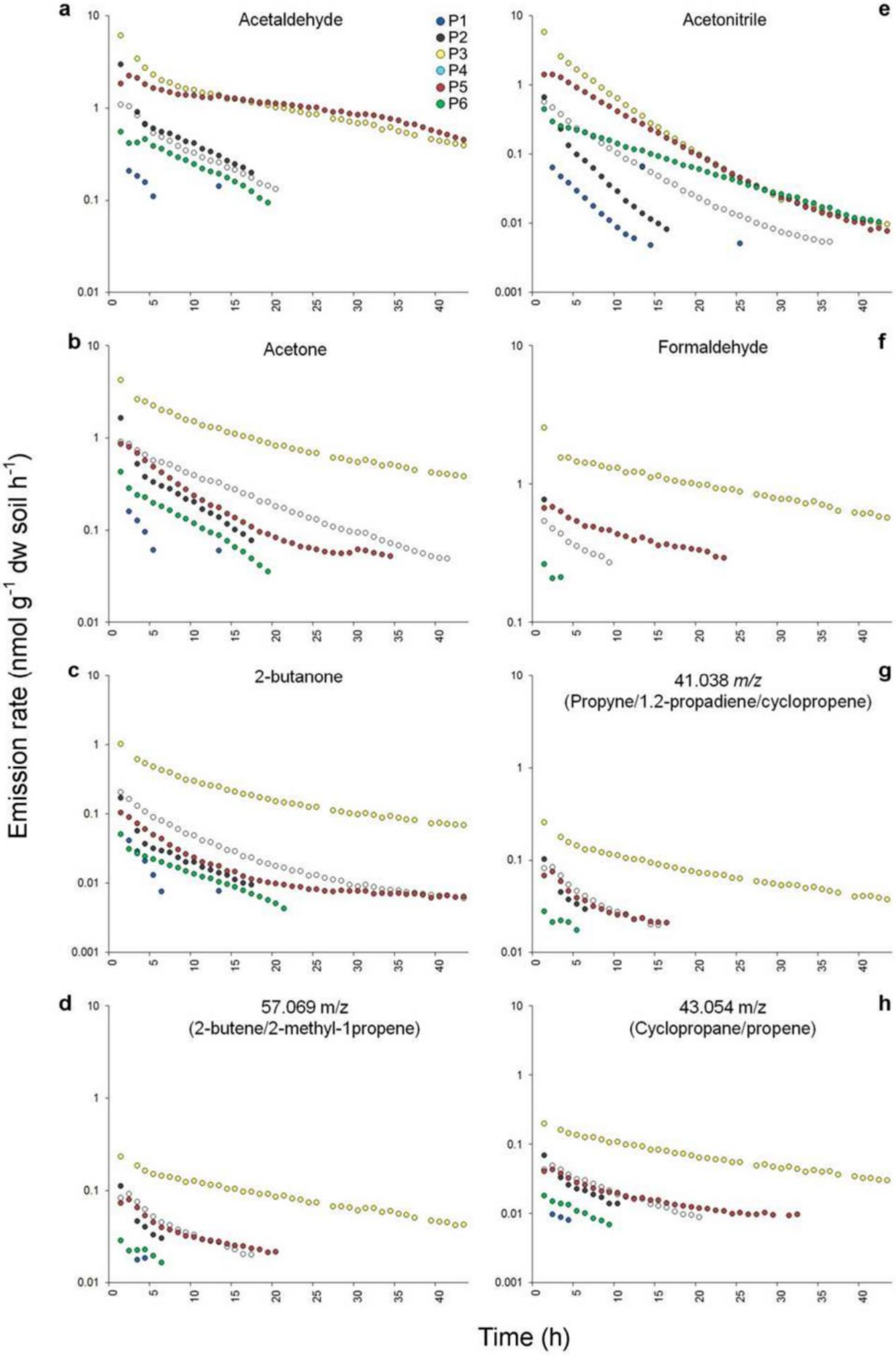

Supplementary Figure 1 │Release of volatiles from thawing permafrost soils. The release of **a**, acetaldehyde **b**, acetone **c**, 2-butanone **d**, 57.069 *m/z* **e**, acetonitrile f, formaldehyde **g**, 41.038 *m/z* and **h**, 43.054 *m/z* across 43 hours is shown for six permafrost soils collected on Disko Island, Greenland. Possible compounds are shown in parentheses. *m/z*=mass to charge ratio;dw=dry weight.

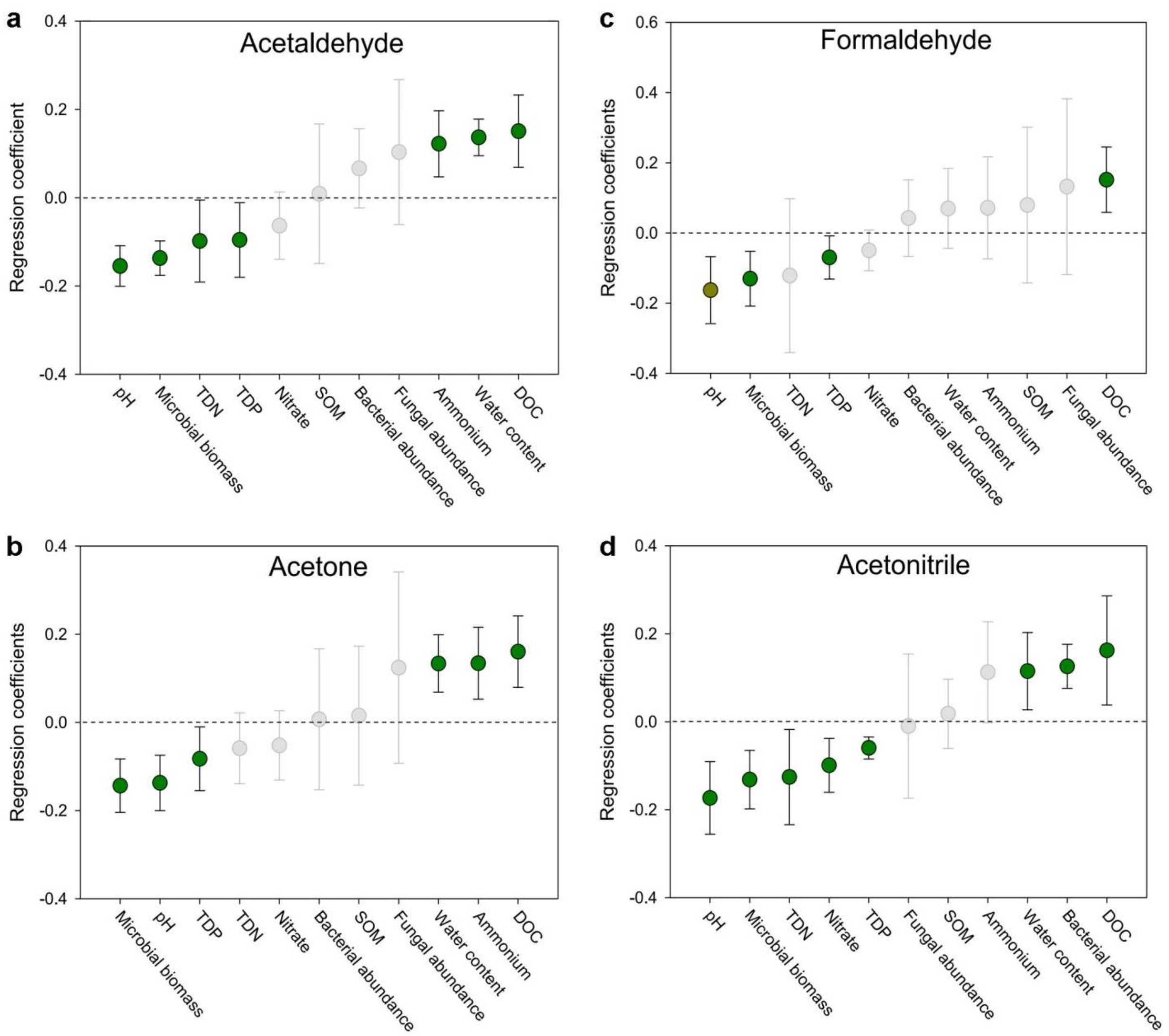

Supplementary Figure 2 │Correlation between permafrost soil characteristics and BVOC release during permafrost thaw. Regression coefficients of partial least squares regression (PLSR) models for the covariance between the measured soil variables and the accumulated release of a) acetaldehyde, b) acetone, c) formaldehyde and d) acetonitrile during the first five hours of permafrost thaw. All models had one PLS component. Positive regression coefficients indicate a positive relationship and negative ones a negative relationship. Error bars show ± confidence intervals (95%) of the regression coefficients. Significant factors are coloured green.

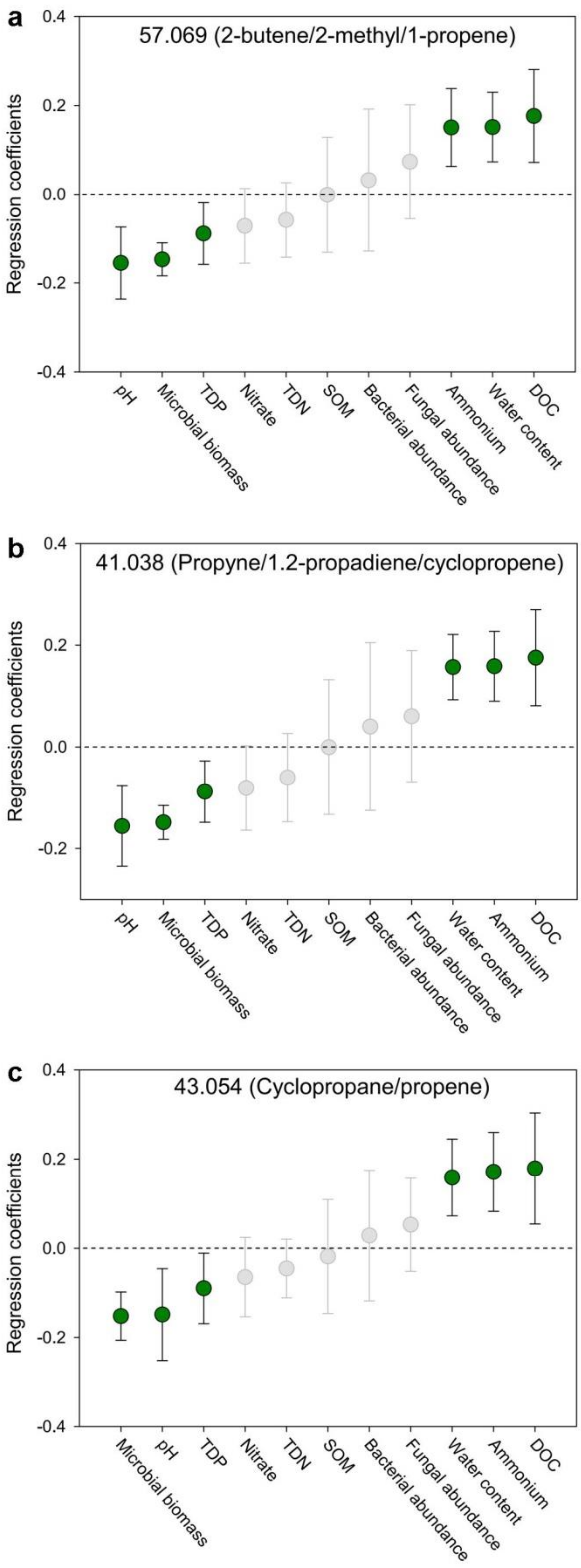

Supplementary Figure 3 │Correlation between permafrost soil characteristics and BVOC release during permafrost thaw. Regression coefficients of partial least squares regression (PLSR) models for the covariance between the measured soil variables and the accumulated release of a) 57.069 m/z, b) 41.038 m/z and c) 43.054 m/z during the first five hours of permafrost thaw. All models had one PLS component. Positive regression coefficients indicate a positive relationship and negative ones a negative relationship. Error bars show ± confidence intervals (95%) of the regression coefficients. Significant factors are coloured green.

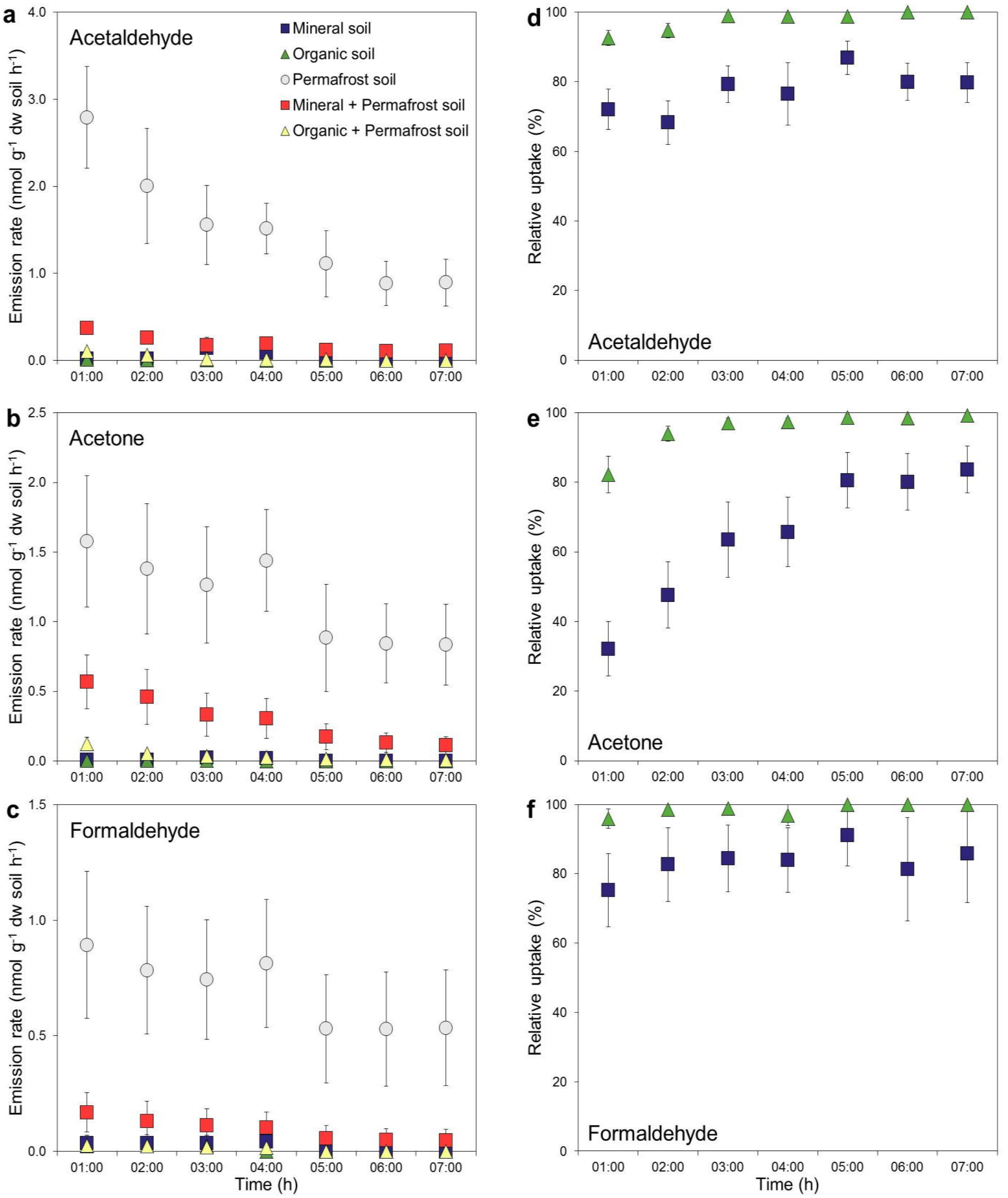

Supplementary Figure 4 │Uptake of volatiles by mineral and organic soil horizons. Emission rate is shown for **a**, acetaldehyde **b**, acetone and **c**, formaldehyde from mineral soil, organic soil, permafrost soil, permafrost + mineral soil and permafrost + organic soil incubated at 6 °C. The relative uptake by mineral and organic soil layers of **d**, acetaldehyde **e,** acetone and **f**, formaldehyde is furthermore shown*.* Possible compounds are shown in parentheses. Error bars show standard error of the mean (n=6). *m*/*z*=mass to charge ratio; dw=dry weight.

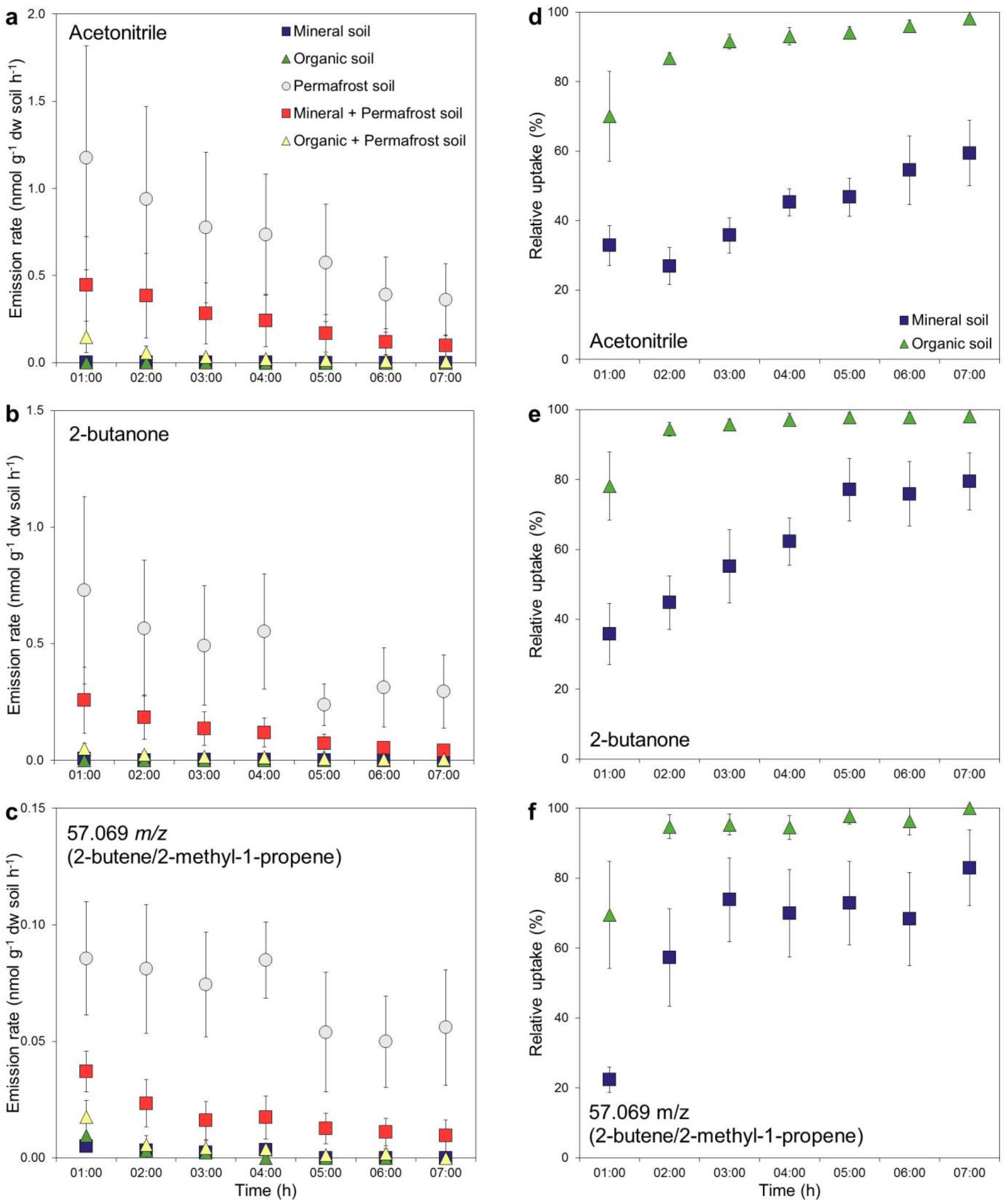

Supplementary Figure 5 │Uptake of volatiles by mineral and organic soil horizons. Emission rate is shown for **a**, acetonitrile **b**, 2-butanone and **c**, 57.069 *m/z* from mineral soil, organic soil, permafrost soil, permafrost + mineral soil and permafrost + organic soil incubated at 6 °C. The relative uptake by mineral and organic soil layers of **d**, acetonitrile **e**, 2-butanone and **f**, 57.069 *m/z* is furthermore shown. Possible compounds are shown in parentheses. Error bars show standard error of the mean (n=6). *m*/*z*=mass to charge ratio; dw=dry weight.

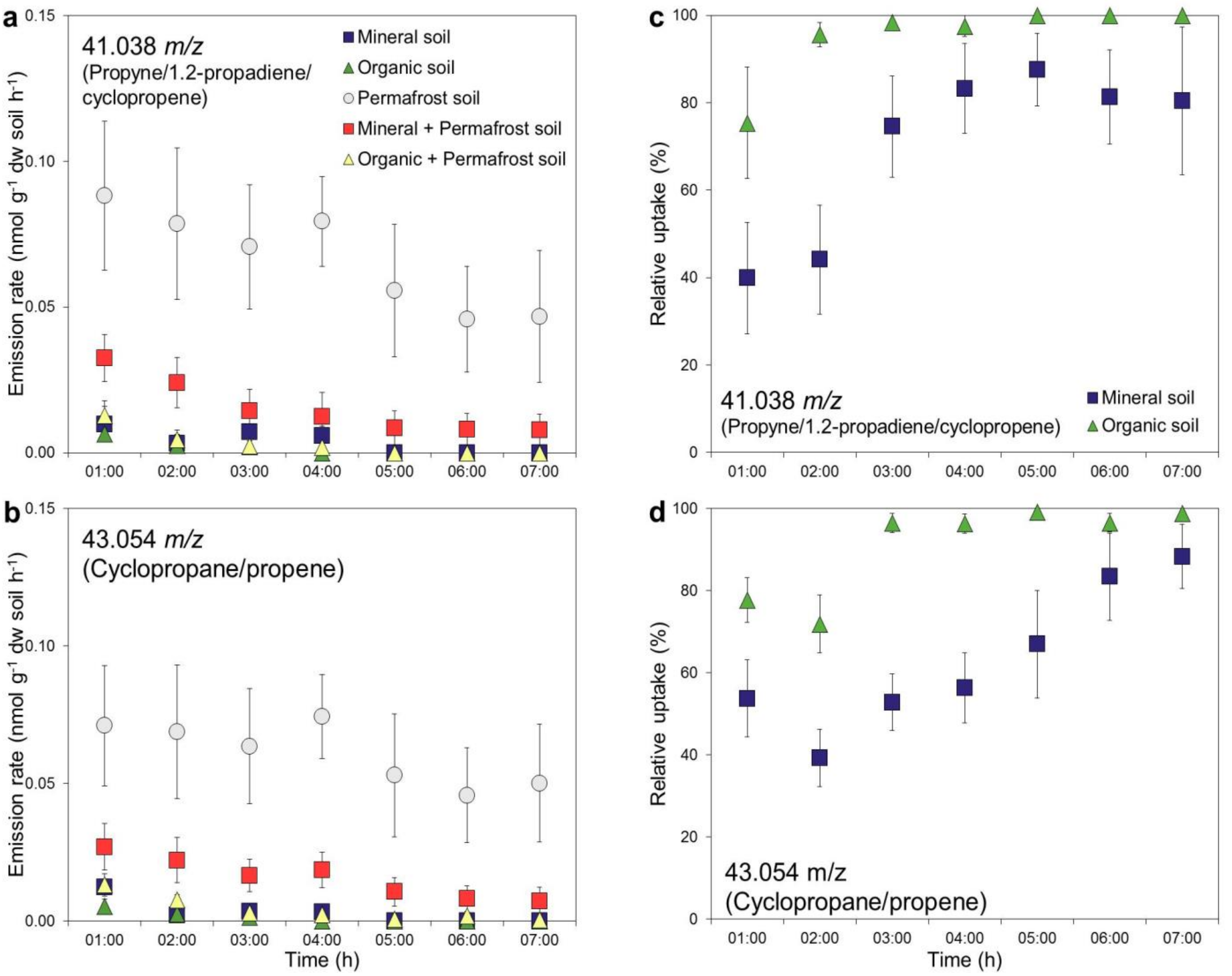

Supplementary Figure 6 │Uptake of volatiles by mineral and organic soil horizons. Emission rate is shown for **a**, 41.038 *m/z* and **b**, 43.054 *m/z* from mineral soil, organic soil, permafrost soil, permafrost + mineral soil and permafrost + organic soil incubated at 6 °C. The relative uptake by mineral and organic soil layers of **c,** 41.038 *m/z* and **d**, 43.054 *m/z* is furthermore shown. Possible compounds are shown in parentheses. Error bars show standard error of the mean (n=6). *m*/*z*=mass to charge ratio; dw=dry weight.

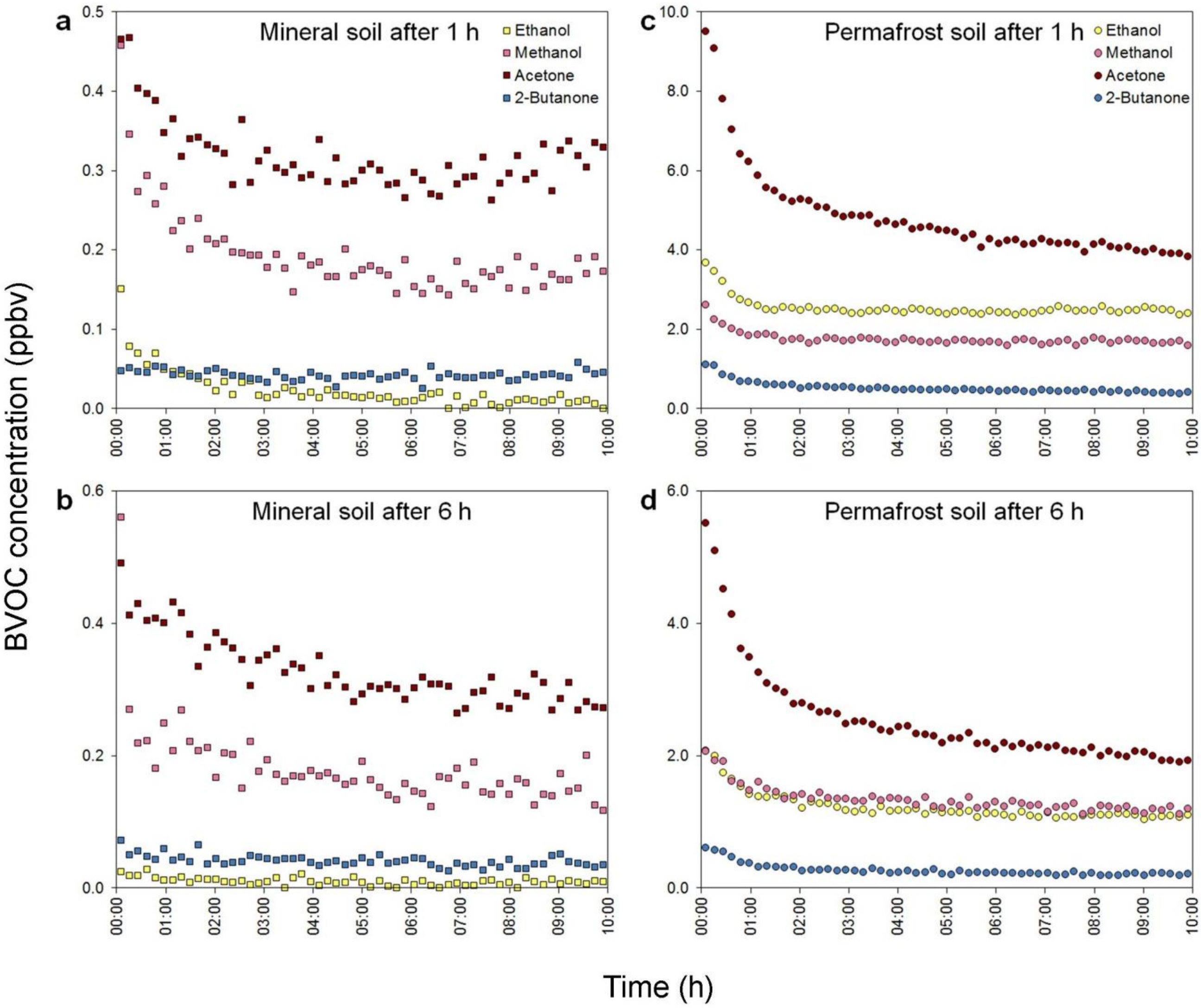

Supplementary Figure 7 │Volatile concentrations in Release experiment. Development of ethanol, methanol, acetone and 2-butanone concentrations during the 10-min long measurement period in **a**, mineral soil after 1 hour **b**, mineral soil after 6 hours **c**, permafrost soil after 1 hour and **d** permafrost soil after 6 hours of incubation. Emission rates were calculated based on average concentrations after 4-8 minutes. Note that the compound concentrations values shown here are not calibrated.

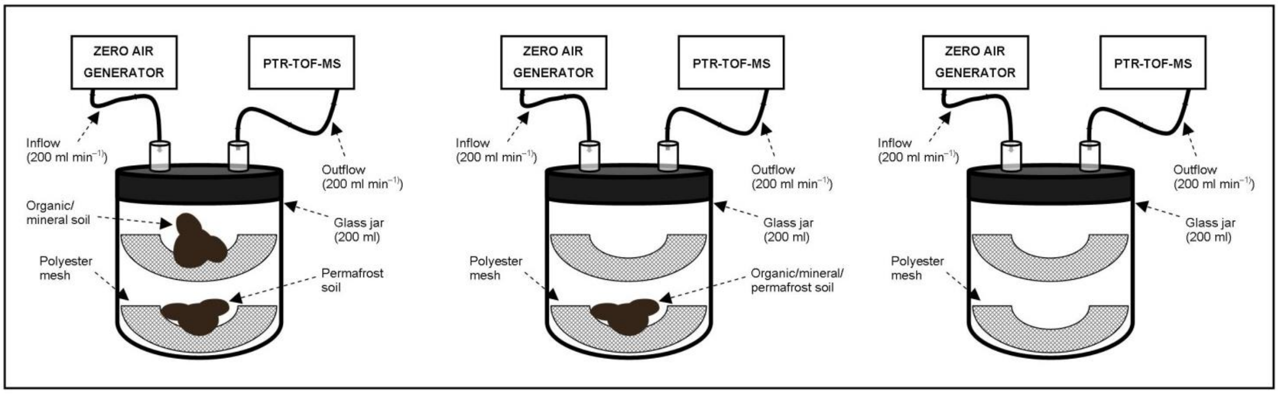

Supplementary Figure 8 │Experimental setup for the Uptake experiment. Schematic figure of the experimental set-up for the uptake experiment showing the different incubation conformations. PTR-TOF-MS=Proton Transfer Reaction – Time Of Flight – Mass Spectrometer.

Supplementary Table 1 │Soil characteristics. Soil parameters measured in organic and mineral horizon bulk soils are presented (± standard error of the mean; *n*=6). Gravimetric water content, soil organic matter, bacterial abundance and fungal abundance were measured on soil samples incubated in the experiments, while other parameters were measured on homogenized bulk soil.

	**Organic horizon**	**Mineral horizon**
pH	5.8 ± 0.04	5.3 ± 0.02
Soil organic matter (%)	40 ± 1.6	12 ± 0.2
Gravimetric water content (%)	48 ± 0.8	31 ± 0.7
DOC (µg g^{-1} dw soil)	186 ± 21	78 ± 12
NO_3-N (ng g^{-1} dw soil)	185 ± 32	56 ± 11
NH_4-N (ng g^{-1} dw soil)	378 ± 38	404 ± 57
TDN (µg N g^{-1} dw soil)	84.4 ± 2.5	21.9 ± 1.4
TDP (µg PO_4 g^{-1} dw soil)	4.3 ± 0.2	0.2 ± 0.03
Bacterial abundance (10^9 x 16S gene copies g^{-1} fw soil)	60 ± 5.6	25 ± 5.4
Fungal abundance (10^5 x ITS2 gene copies g^{-1} fw soil)	379 ± 42	41 ± 1.9
Microbial biomass (µg g-1 dw soil)	1522 ± 47	410 ± 29

DOC=dissolved organic carbon; TDN=total dissolved nitrogen; TDP= total dissolved phosphorus; fw=fresh weight; dw=dry weight.

Supplementary Table 2 │Compound calibration factors. Calibration factors calculated from a calibration gas mixture.

Compound	Mass to charge ratio	Calibration factor
Formaldehyde	31.018	9.03
Methanol	33.033	3.23
Acetonitrile	42.034	1.12
Acetaldehyde	45.033	1.1
Ethanol	47.049	19.85
Acetone	59.049	1.04
Isoprene	69.07	2.41
Crotonaldehyde	71.049	0.97
2-butanone	73.064	1.09
Benzene	79.054	1.79
Monoterpenes-1	81.07	3.45
Toluene	93.07	1.48
O-xylene	107.085	1.56
Chlorobenzene	113.023	2.26
Monoterpenes-2	137.133	3.86
1,2-dichlorobenzene	146.97	2.32
1,2,4-trichlorobenzene	180.991	5.62

Large calibration factors may result from four factors: (i) the reaction rate constant is significantly lower than the default value, (ii) the molecule fragments during ionization, (iii) scattered stable isotope distribution (e.g. the total of the major peak is less than 60% of total signal), and (iv) significant backward reaction (de-protonation) in the case of formaldehyde.
