# Supplementary material for: Biogenic volatile release from permafrost thaw is determined by the soil microbial sink
Source: Nat Commun. 2018 Aug 24;9:3412. doi: 10.1038/s41467-018-05824-y (PMC6109083; doi:10.1038/s41467-018-05824-y)
Supplement: Supplementary file 3 — Description of Additional Supplementary Files [file 41467_2018_5824_MOESM3_ESM.pdf]

Description of Additional Supplementary Files

File Name: Supplementary Data 1

Description: List of volatiles measured. Complete list of average emission rates (picomol g^{-1} dry weight soil h^{-1}) of the mass to charge ratios detected in the Release and Uptake experiments (mean ± SEM, n=6). Δ mDa indicates the difference between the possible empirical formula mass and the measured mass. Average emission rates are calculated based on the entire incubation period. nd=not detected
