# Supplementary material for: Biogenic volatile release from permafrost thaw is determined by the soil microbial sink
Source: Nat Commun. 2018 Aug 24;9:3412. doi: 10.1038/s41467-018-05824-y (PMC6109083; doi:10.1038/s41467-018-05824-y)
Supplement: Supplementary file 2 — Peer Review [file 41467_2018_5824_MOESM2_ESM.pdf]

Reviewers' comments:

Reviewer #1 (Remarks to the Author):

The paper by Kramshøj et al. is well written, methodologically sound, and the conclusions are almost fully supported by the data. I read the paper with great interest and am very excited about the authors' highly novel findings which have important implications for global change impacts. This is an important work utilizing the state-of-the-art analytical instrumentation to address the big science questions about VOC emissions from thawing permafrost. I feel that the manuscript should be of interest for the broad audience of Nature Communications and for these reasons I would strongly recommend publication after considering these relatively minor comments/suggestions below:

VOC uptake
I find it intriguing that the active soil layer is the sink for the occluded microbial metabolites and I generally like the uptake experiment. In line 112 it is written: „The large BVOC uptake observed could therefore be a result of microbial consumption". I think you might be right that many different species of microbes would not mind taking up these compounds, but do you neglect to notice that the soil itself may have a huge active surface which might have an adsorption efficiency comparable to an activated charcoal? Although it might seem intuitive, I think it is difficult to get fully convinced how much would be microbial uptake vs the bulk soil itself. If you have a charcoal or a high surface catalyst would it not be expected to convert the isotopic methanol and ethanol to CO_2 and H_2O? Have you done that experiment (would need a fresh charcoal)? Could it be that you are finding a decline in uptake because of the soil saturation and breakthrough?

I realize that my hypothesis can possibly be easily rejected but what I would like is to inspire authors' thinking to convince themselves that this is really related mostly to the microbial uptake and not a chemico-physical uptake. An easy experiment would be to repeat the same C14 study simultaneously on the sterilized and natural soil and maybe this has already been done. Could comparing active surface to permafrost soils be misleading if the thawing occurred a different number of days before the experiment, so the volume could already have a chance to get saturated by methanol and ethanol (which are typically abundant gases in the atmosphere) and hence might not show significant uptake?

If it is not possible to unambiguously determine the microbial vs nonmicrobial uptake, an alternative simple solution would be to tone down the conclusions about microbial active source by more balanced possibility of nonmicrobial sinks. Comparing identical samples half of which underwent sterilization would be very confirmative. Such a sterilization would be difficult to achieve without influencing the chemistry but I would expect that nonmicrobial soil would also be a large sink for the organics and likely a "catalyst" due to the presence of metals catalyzing convertion of organics to CO_2 and H_2O. Yet another possibility would be to compare with the genomic analysis of microbial diversity if it is consistent with Methylotrophic species in high methanol uptake samples. Interestingly, the authors write that the presence of microbes did not sometimes result in an uptake which is explained by a different microbial activity or metabolic capability (L149-152). I would suspect this may have as well been due to a longer time after thawing compared to other samples, allowing for reaching saturated concentration of some of the gases, but I wonder if the authors have means for excluding this possibility.

Insights into microbial life
Would it be possible to estimate how much anaerobic vs aerobic life is/was there based on the comprehensive composition of organic gases? It seems that the focus is largely on the most abundant compounds but the less abundant ones could provide more specific information about the processes. I would suspect there must be redox processes regulated by the availability of electron acceptors and there must be many specific organic tracers which could tell us a lot about what has been degraded (e.g. volatile conversion products of celulose, lignin, pectin, proteins,

fatty acids, etc.).

Micorbial vs nonmicrobial chemistry
Organic mass and plant residues might contain lignocellulosic biopolymers. While I have no doubt that these also can be degraded by microbes, another possibility to consider is that there might be an autocatalytic cycle mediated by acetic acid and acidic pH (the SI table shows the soils were mildly acidic). In these conditions the degradation of these polymers similarly as what is happening to wood degradation might lead to acetic acid (further lowering pH and acting as an autocatalyst) and possibly a bunch of furanoids and benzenoids which I am curious if the authors have observed. My question is whether it is justified to fully attribute all these gases to the microbial life or should it be at least described with a greater caution that some of the compounds could also come from nonmicrobial processes such as slow chemical degradation? Although I do not suggest that the author inferences must necessarily be incorrect, I would think that the latter chemical process could deserve a greater emphasis in the paper.

Complexity of organic ions
It seems that the authors have correctly used the approaches included in ptrwid which is in my opinion currently the most advance state-of-the-art approach to embrace the complexity of the detected VOC ions. Yet, the focus of the paper seems to be mostly on a few prominent compounds. I agree that the massive emissions of ethanol are definitely important for the atmosphere, but there could be many reactive less abundant compounds. I think that the remaining hundreds of compounds could also be very interesting for the readers, but if it is not possible to present them in a meaningful simple way in this paper, I would recommend a table of detected ions in the SI, which is becoming somewhat a common practice in many PTRTOF manuscripts. For example, a table of emission factors for all the VOC ions could be useful for comparisons with future studies.

Quantification of uncertainties
I really appreciate that the abundant compounds have all been calibrated by the authentic standard, which shows high technical quality of the data and I assume the accuracy is very high for these compounds. I would like to suggest to mention the actual percentage uncertainty of different groups of compounds (calibrated and predicted from collision rate theory). Alternatively one could add range bounds on the reported emission rates.

Technical
L46. References need reformating.
Figure 1. Explain what p1-p6 refer to or change into more meaningful labels.

Reviewer #2 (Remarks to the Author):

The authors report the release of significant amounts of biogenic volatile organic compounds (BVOC) from thawing permafrost, which adds an important new perspective on impacts from Arctic warming and expected widespread permafrost thaw this century. They also demonstrate that two of the most abundant BVOCs, methanol and ethanol, are substantially adsorbed or mineralized by active layer soils, constraining the potential net emissions of BVOCs to the atmosphere. This work addresses an aspect of the Arctic carbon cycle that is not yet depicted in most conceptual models, let alone quantitative process-rich models and simulations.

The authors need to address several technical and conceptual issues outlined below to make this work conclusive. More information is required to explain the quantitative analysis used to analyze ethanol among mixed BVOCs. There is substantial variability in BVOC emission rates from the six tested samples: can that variability be explained by any combination of the permafrost properties?

The source of BVOCs in permafrost needs to be clarified -are they frozen in permafrost waiting to be released, or are they generated by rapid microbial degradation of thawed organic matter?

Lines 63-68. Were only molecular ions [MH+] measured? Quantifying ethanol using the proton transfer reaction-time of flight-mass spectrometry (PTR-MS) method applied here is quite difficult. The authors report a very high calibration factor for ethanol in Suppl. Table 4, consistent with low response factors measured by others. Such low sensitivities magnify the impacts of any variation in ethanol ion counts. In PTR-MS systems, ethanol appears to react with hydronium ions to form a variety of ethanol-related ions and clusters (see for example Sémon et al. J. Mass Spectrom. (2018) 53:65). Since there is no separation step upstream in this PTR-MS method, should we be concerned about artifacts and significant deviations in response factors from the pure calibration gas standard? The authors need to demonstrate that matrix effects and high ethanol concentrations do not bias the analysis, otherwise the reported results will not be reliable. Perhaps a standard addition experiment adding an appropriate dilution of calibration gas to a relevant environmental sample matrix would build confidence in the method.

Line 287. Can the PTR-TOF-MS instrument used here routinely distinguish between ethanol and formic acid molecular ions based on the reported mass resolution?

Lines 76-77, Figures 1 & S1. There is substantial variation in emission rates of ethanol, methanol and other BVOCs among the six soil samples tested. What could explain the variation?

Lines 79-86. This work begs the question whether BVOCs are trapped in the frozen permafrost and progressively released in this experiment, or whether they are rapidly produced by decomposition processes in the thawed soils. The authors offer a kinetic argument against the decomposition model but provide little data. In this system, would it be possible to rapidly extract alcohols and BVOCs from under acidified conditions that inhibit enzymatic activity for comparison?

Specific comments

Lines 39-49. The point and organization of this paragraph need clarification. Is it intended to introduce the problem of permafrost thaw or microbial activity at low temperatures?

Lines 72-73. Several different enzymes of central metabolism can produce the precursors of ethanol. Not necessary to list enzymes here.

Lines 89-94. The manuscript extrapolates potential ethanol emissions from highly variable measurements of six permafrost samples from one site. As the authors note, such linear extrapolations are challenging. There are vast differences in permafrost composition across the Arctic, so more sophisticated modeling will be required to address the variability observed here and simulate potential emissions. Without further support, this paragraph should be omitted from the present work.

Lines 110-112. Methanol can also be consumed by some acetogens and methanogens.

Figure 2. The letters of panels in this figure do not match the legend. If the error bars represent the standard error of the mean, why are they not symmetric with the mean values plotted as symbols?

Figure 3 illustrating mineralization results from the addition of radiolabeled methanol and ethanol is a valuable addition to this manuscript. Good controls to distinguish adsorption from assimilation of unrecovered 14C-ethanol. Nice work.

Lines 165-177 A short description of the permafrost samples is needed here. Average organic content? Water? Oxic or anoxic?

Lines 189-194. Were the active layer organic and mineral soils taken from the same site as the cores listed in the site description? If different, what is the rationale for mixing layers?

Reviewer #3 (Remarks to the Author):

The topic of the research is highly reelvant and timely.
The manuscript is written in a very clear and straightforward way. Statistics seems to be ok, and the results, albeit little surprising, add to the understanding.

I am not expert in the physical aspects of the measurements, I am just wondering of polyethylen as used for the bags does not retain any of the VOCs (memory effects) and thus my make measurements inaccuarate.

you have used the term 'active layer soil' in some places, I would find a term like 'soil from the active layer' more appropriate, nbut I guess this is a matter of taste.

line 102 omit comma after showed

Dear Reviewers,

We would like to thank for the thorough and insightful comments on our manuscript. We have incorporated all the relevant suggested changes and responded to all the comments below. Our answers are shown in grey background and the line numbers refer to the revised version of the manuscript.

Reviewer #1

The paper by Kramshøj et al. is well written, methodologically sound, and the conclusions are almost fully supported by the data. I read the paper with great interest and am very excited about the authors' highly novel findings which have important implications for global change impacts. This is an important work utilizing the state-of-the-art analytical instrumentation to address the big science questions about VOC emissions from thawing permafrost. I feel that the manuscript should be of interest for the broad audience of Nature Communications and for these reasons I would strongly recommend publication after considering these relatively minor comments/suggestions below:

VOC uptake

I find it intriguing that the active soil layer is the sink for the occluded microbial metabolites and I generally like the uptake experiment. In line 112 it is written: „The large BVOC uptake observed could therefore be a result of microbial consumption". I think you might be right that many different species of microbes would not mind taking up these compounds, but do you neglect to notice that the soil itself may have a huge active surface which might have an adsorption efficiency comparable to an activated charcoal? Although it might seem intuitive, I think it is difficult to get fully convinced how much would be microbial uptake vs the bulk soil itself. If you have a charcoal or a high surface catalyst would it not be expected to convert the isotopic methanol and ethanol to CO2 and H2O? Have you done that experiment (would need a fresh charcoal)? Could it be that you are finding a decline in uptake because of the soil saturation and breakthrough?

We are not aware of natural abiotic processes in soil that may convert methanol and ethanol to CO_2 and water. We do agree that adsorption may occur, and even though the mineralization experiments show that fast microbial degradation of the BVOCs occurs in the soil, this does not mean that adsorption to the soil cannot occur simultaneously. However, the relative uptake increases with time (there is no decline in uptake as the Reviewer states). Such an increase would not be expected if adsorption was the major removal process. Actually, since emission rates decrease over time, you would expect a decrease in uptake or even a net release, since adsorption to soil would normally be reversible for volatile compounds such as methanol and ethanol in contrast to microbial degradation. We have improved the discussion as follows: "This uptake could be caused by BVOC sorption to soil particles and organic material, dissolution in the water phase for hydrophilic compounds or microbial consumption. All three processes occur to some extent, but our PTR-TOF data suggest that microbial uptake was the major removal process. We observed that the relative BVOC uptake increased with time, and that owes support to biotic rather than physico-chemical processes. In physico-chemical processes the relative uptake would be expected to decrease rather than increase, as adsorption or dissolution would likely be reversible in contrast to microbial degradation of the compounds." (l. 165-171)

I realize that my hypothesis can possibly be easily rejected but what I would like is to inspire authors' thinking to convince themselves that this is really related mostly to the microbial uptake and not a chemico-physical uptake. An easy experiment would be to repeat the same C14 study simultaneously on the sterilized and natural soil and maybe this has already been done. Could comparing active surface to permafrost soils be misleading if the thawing occurred a different number of days before the experiment, so the volume could already have a chance to get saturated by methanol and ethanol (which are typically abundant gases in the atmosphere) and hence might not show significant uptake?

If it is not possible to unambiguously determine the microbial vs nonmicrobial uptake, an alternative simple solution would be to tone down the conclusions about microbial active source by more balanced possibility of nonmicrobial sinks. Comparing identical samples half of which underwent sterilization would be very confirmative. Such a sterilization would be difficult to achieve without influencing the chemistry but I would expect that nonmicrobial soil would also be a large sink for the organics and likely a "catalyst" due to the presence of metals catalyzing convertion of organics to CO2 and H2O.

Thank you for the comment. We fully agree in this concern and have given it much thought when planning and conducting the experiment. As a matter of fact, we had done sterilization experiments as the reviewer suggested. In these mineralization experiments we tested whether sterilized soil without microbial activity would convert methanol and ethanol into CO_2. This was done using radiocarbon labelled methanol and ethanol. We observed that with natural microbial community (unsterilized samples) about 95% of the added 14C-methanol and 15% of the added ^{14}C-ethanol was transformed into ^{14}C-CO_2. In contrast, sterilized soil did not convert the added methanol and ethanol to CO2, which indicates that this conversion does not take place without living microbial community.
We realize, that this was not very clearly described. We have therefore added the following sentences to the manuscript:
Results (L. 131-133): "To test if the observed mineralization could be due to abiotic degradation of ethanol and methanol, we included soil samples sterilized by autoclaving in the experiment. In the sterilized samples, only 1% of the methanol and none of the ethanol was mineralized to CO2 after 144 hours."
Discussion (L. 174-175): "This conversion did not take place in sterilized soil samples indicating that it was in fact a result of microbial activity."

Yet another possibility would be to compare with the genomic analysis of microbial diversity if it is consistent with Methylotrophic species in high methanol uptake samples. Interestingly, the authors write that the presence of microbes did not sometimes result in an uptake which is explained by a different microbial activity or metabolic capability (L149-152). I would suspect this may have as well been due to a longer time after thawing compared to other samples, allowing for reaching saturated concentration of some of the gases, but I wonder if the authors have means for excluding this possibility.

A genomic analysis of microbial diversity could be done, but it would only show that the potential for degradation is present. The mineralization experiments already included are better; showing that fast mineralization is actually occurring (and to our knowledge this must be microbial, which is further supported by no mineralization in the sterile controls).

The difference between uptake rates in some vs. other samples was simply the difference between the active layer soil samples as compared with the permafrost samples. The lines 149-152 in the original

manuscript refer to the low uptake in *all* the samples from permafrost. We have now added some discussion (L. 191-195) on this: "As opposed to the active layer soils, degradation of ethanol and methanol in permafrost did not start until 72-144 hours of incubation. This is in agreement with previous experiments with permafrost soil showing that microbial respiration starts to increase three days after thaw and peaks after two weeks. The initial lag phase can most likely be explained by that the degrader organisms needed some time to adjust to the new conditions and become active or that microbial growth was needed to obtain the increase in activity."

Insights into microbial life

Would it be possible to estimate how much anaerobic vs aerobic life is/was there based on the comprehensive composition of organic gases? It seems that the focus is largely on the most abundant compounds but the less abundant ones could provide more specific information about the processes. I would suspect there must be redox processes regulated by the availability of electron acceptors and there must be many specific organic tracers which could tell us a lot about what has been degraded (e.g. volatile conversion products of celulose, lignin, pectin, proteins, fatty acids, etc.).

Thank you for pointing out that we had not clearly enough explained that the permafrost samples had high enough water content to cause anoxic conditions upon thaw due to water saturation of the soil. As a matter of fact, methanol and ethanol are typical indicators of anaerobic microbial activity, which appears to have dominated in the studied permafrost. We have now clarified these issues in the revised manuscript: "Due to the high water content, the permafrost samples have most likely contained anaerobic microsites during the experiments." (L. 88-90). Based on the partial least squares regression analysis we conducted, the most emitted BVOCs had similar correlations with the measured soil characteristics as ethanol and methanol suggesting that they originated from the same processes.

We have gone through the released compounds to identify specific biomarkers. We found that the presence of acetonitrile could indicate existence of wildfires in the area where the soils were sampled. Several of the benzenoids released in this experiment could be related to lignin degradation, but we have not been able to connect other compounds to specific soil processes. The use of VOCs as biomarkers is indeed an interesting question, but we have not gone further into it as we believe it is beyond the scope of the present study.

Micorbial vs nonmicrobial chemistry

Organic mass and plant residues might contain lignocellulosic biopolymers. While I have no doubt that these also can be degraded by microbes, another possibility to consider is that there might be an autocatalytic cycle mediated by acetic acid and acidic pH (the SI table shows the soils were mildly acidic). In these conditions the degradation of these polymers similarly as what is happening to wood degradation might lead to acetic acid (further lowering pH and acting as an autocatalyst) and possibly a bunch of furanoids and benzenoids which I am curious if the authors have observed. My question is whether it is justified to fully attribute all these gases to the microbial life or should it be at least described with a greater caution that some of the compounds could also come from nonmicrobial processes such as slow chemical degradation? Although I do not suggest that the author inferences must necessarily be incorrect, I would think that the latter chemical process could deserve a greater emphasis in the paper.

We agree with the reviewer that we cannot fully justify claiming that all produced gases originate from microbial activity, and we have now opened up the discussion to include abiotic processes. We have therefore added the following sentences in the discussion: “Furthermore, compounds such as ethanol, methanol and acetone can be produced in non-enzymatic thermochemical Maillard reactions. Such reactions are, however, strongly temperature-dependent, and due to the low incubation temperature used in our experiments, most likely only a minor part of the observed BVOC release is derived from abiotic processes.” (L. 150-153).

Complexity of organic ions

It seems that the authors have correctly used the approaches included in ptrwid which is in my opinion currently the most advance state-of-the-art approach to embrace the complexity of the detected VOC ions. Yet, the focus of the paper seems to be mostly on a few prominent compounds. I agree that the massive emissions of ethanol are definitely important for the atmosphere, but there could be many reactive less abundant compounds. I think that the remaining hundreds of compounds could also be very interesting for the readers, but if it is not possible to present them in a meaningful simple way in this paper, I would recommend a table of detected ions in the SI, which is becoming somewhat a common practice in many PTRTOF manuscripts. For example, a table of emission factors for all the VOC ions could be useful for comparisons with future studies.

We fully agree. In fact, tables of all the detected VOC ions and their emission rates were/are presented in the Supplementary Dataset as Supplementary Table 3. As this was a separate file from the rest of the Supplementary Information, it was easy to lose or overlook. The Supplementary Table 3 was earlier only referred to in the Methods, but we have now added a reference to it in the Results instead: “A complete list of released ions is shown in Supplementary Table 1.” (l. 79-80).

Quantification of uncertainties

I really appreciate that the abundant compounds have all been calibrated by the authentic standard, which shows high technical quality of the data and I assume the accuracy is very high for these compounds. I would like to suggest to mention the actual percentage uncertainty of different groups of compounds (calibrated and predicted from collision rate theory). Alternatively one could add range bounds on the reported emission rates.

Thank you for the suggestion. We have added the following sentence in the Methods (l. 356-358): “The accuracy for estimation of the concentrations of the compounds without specific calibration standards was estimated to be +/- 40 % for the ions with *m/z* below 150 and +/- 60 % for the ions with *m/z* above.”

Technical

L46. References need reformating.

Figure 1. Explain what p1-p6 refer to or change into more meaningful labels.

We have checked through and corrected references.
We have added an explanation to what P1-P6 means.

Reviewer #2

The authors report the release of significant amounts of biogenic volatile organic compounds (BVOC) from thawing permafrost, which adds an important new perspective on impacts from Arctic warming and expected widespread permafrost thaw this century. They also demonstrate that two of the most abundant BVOCs, methanol and ethanol, are substantially adsorbed or mineralized by active layer soils, constraining the potential net emissions of BVOCs to the atmosphere. This work addresses an aspect of the Arctic carbon cycle that is not yet depicted in most conceptual models, let alone quantitative process-rich models and simulations.

The authors need to address several technical and conceptual issues outlined below to make this work conclusive. More information is required to explain the quantitative analysis used to analyze ethanol among mixed BVOCs. There is substantial variability in BVOC emission rates from the six tested samples: can that variability be explained by any combination of the permafrost properties? The source of BVOCs in permafrost needs to be clarified -are they frozen in permafrost waiting to be released, or are they generated by rapid microbial degradation of thawed organic matter?

We thank the reviewer for the thorough work and constructive criticism. We have considered all of these issues and will answer to the specific comments below.

Lines 63-68. Were only molecular ions [MH+] measured? Quantifying ethanol using the proton transfer reaction-time of flight-mass spectrometry (PTR-MS) method applied here is quite difficult. The authors report a very high calibration factor for ethanol in Suppl. Table 4, consistent with low response factors measured by others. Such low sensitivities magnify the impacts of any variation in ethanol ion counts. In PTR-MS systems, ethanol appears to react with hydronium ions to form a variety of ethanol-related ions and clusters (see for example Sémon et al. J. Mass Spectrom. (2018) 53:65). Since there is no separation step upstream in this PTR-MS method, should we be concerned about artifacts and significant deviations in response factors from the pure calibration gas standard? The authors need to demonstrate that matrix effects and high ethanol concentrations do not bias the analysis, otherwise the reported results will not be reliable. Perhaps a standard addition experiment adding an appropriate dilution of calibration gas to a relevant environmental sample matrix would build confidence in the method.

Sémon et al., 2018, DOI: 10.1002/jms.4036 measured a 10% ethanol water solution, so they got much higher counts of ethanol ions and the associated matrix effects (cluster combinations of ethanol and water). In our experiments the ethanol concentrations were much lower and we did not observe significant amounts of ethanol-ethanol or ethanol-water clusters. For our experiments, only the formation rate of dehydrated ethanol ($C_2H_5^+$) controls the sensitivity of ethanol. This is very sensitive to E/N and humidity, maybe also pressure and temperature of the drift tube. It is therefore important that the calibrations are done under the same conditions as the real samples. In our experiments, we performed calibrations immediately before, during and after the experiments in conditions similar to those of the incubations. The absolute humidity in the incubation air and the calibration air was approximately 7.3 and 10.4 g m^{-3}, respectively.

Line 287. Can the PTR-TOF-MS instrument used here routinely distinguish between ethanol and formic acid molecular ions based on the reported mass resolution?

At a resolution of 3000-4000 (FWHM) the ethanol peak is 0.012-0.016 Th broad. The difference between ethanol and formic acid is 0.036. So, the two peaks are fully separated and can be clearly distinguished.

Lines 76-77, Figures 1 & S1. There is substantial variation in emission rates of ethanol, methanol and other BVOCs among the six soil samples tested. What could explain the variation?

This is a very good point. We have now conducted statistical analyses to assess what could explain the variation in emission rates from permafrost for the 10 most emitted compounds. This was done by partial least squares regression analysis, which is a flexible multivariate regression technique suitable to analyze for covariance between a dependent variable (e.g. ethanol emission) and several potentially correlated explanatory variables. The regression coefficients between the variables characterizing the soil and ethanol & methanol release have been presented as Fig. 2 and those for the other most emitted BVOCs as Supplementary Fig. 3 and 4. Corresponding text has been added to Methods (L. 390-396), Results (L. 90-98) and Discussion (L. 155-160). We found that the emissions of ethanol, methanol and most other compounds positively correlated with the dissolved organic carbon and ammonium concentrations as well as the water content of permafrost (Fig. 2, Supplementary Fig. 3 and 4). The variables showing strongest negative correlations with the emission rates were pH, microbial biomass, and water-extractable dissolved phosphorus concentration.

Lines 79-86. This work begs the question whether BVOCs are trapped in the frozen permafrost and progressively released in this experiment, or whether they are rapidly produced by decomposition processes in the thawed soils. The authors offer a kinetic argument against the decomposition model but provide little data. In this system, would it be possible to rapidly extract alcohols and BVOCs from under acidified conditions that inhibit enzymatic activity for comparison?

We agree that the origin of the released BVOCs can be further discussed, and we have now expanded the discussion on this (L. 138-145): "This rapid release and the subsequent decline in BVOC release has two potential explanations: previous production processes may have occurred in the frozen permafrost soil, slowly causing a buildup of immobilized BVOCs that were now released. Alternatively, the turnover of a limited pool of labile carbon made available upon thaw, could cause an initially high microbial fermentation rate and a release of trace gases that decreases over time. In Arctic active layer soils, a large ethanol production has been shown to correlate with a depletion of labile carbon made available upon thaw. However, the rapid appearance of the release peak at low temperature (6 °C) points to a release of previously trapped gasses rather than post-thaw microbial production, as earlier shown for methane."

Specific comments

Lines 39-49. The point and organization of this paragraph need clarification. Is it intended to introduce the problem of permafrost thaw or microbial activity at low temperatures?

When restructuring the manuscript to Nature Communications format, this paragraph has now been split into two paragraphs, the first one of which introduces microbial activity at low temperatures and the second the permafrost thaw issue.

Lines 72-73. Several different enzymes of central metabolism can produce the precursors of ethanol. Not necessary to list enzymes here.

As suggested, we have deleted the last part of the sentence mentioning any specific enzymes.

Lines 89-94. The manuscript extrapolates potential ethanol emissions from highly variable measurements

of six permafrost samples from one site. As the authors note, such linear extrapolations are challenging. There are vast differences in permafrost composition across the Arctic, so more sophisticated modeling will be required to address the variability observed here and simulate potential emissions. Without further support, this paragraph should be omitted from the present work.

We have considered whether or not to include this rough extrapolation, and have decided to agree with the reviewer and remove the paragraph from the paper. We have added a sentence on lines 212-214 to acknowledge that the highest emission rates were that high that the emission should be considered: "As the maximum emission rates observed suggest that permafrost may be a significant source of BVOCs to the Arctic atmosphere, it is important to further address the processes releasing and consuming BVOCs in soil in future studies."

Lines 110-112. Methanol can also be consumed by some acetogens and methanogens.

During the revision of the manuscript the sentence has been removed.

Figure 2. The letters of panels in this figure do not match the legend. If the error bars represent the standard error of the mean, why are they not symmetric with the mean values plotted as symbols?

Thank you, the letters of panels in this figure (now Figure 3) have been corrected.

The error bars do represent standard error of the mean and are symmetric. We have chosen only to show the positive direction in order to increase legibility of the figure, but we had not clearly stated this in the figure legend. This mistake has now been clarified and the figure legend corrected.

Figure 3 illustrating mineralization results from the addition of radiolabeled methanol and ethanol is a valuable addition to this manuscript. Good controls to distinguish adsorption from assimilation of unrecovered 14C-ethanol. Nice work.

Thank you for the kind words. We were very happy to get the technique working and being able to test our hypothesis.

Lines 165-177 A short description of the permafrost samples is needed here. Average organic content? Water? Oxic or anoxic?

We have pulled the Table reporting soil characteristics from the supplementary to the main text as Table 2. The following sentence has been added on L. 87-90: "The organic matter content and the gravimetric water content of the permafrost samples averaged 28% and 48%, respectively, with considerable variation between individual samples (Table 2). Due to the high water content, the permafrost samples have most likely contained anaerobic microsites during the experiments."

Lines 189-194. Were the active layer organic and mineral soils taken from the same site as the cores listed in the site description? If different, what is the rationale for mixing layers?

All the samples originated from the same area and thus represent West Greenlandic permafrost. The sampling site has been described on lines 221-230 and the sampling of soils on lines 236-249. The active layer soil cores were divided into organic and mineral horizons. The description has been clarified.

Reviewer #3

The topic of the research is highly reelvant and timely.
The manuscript is written in a very clear and straightforward way. Statistics seems to be ok, and the results, albeit little surprising, add to the understanding.

I am not expert in the physical aspects of the measurements, I am just wondering of polyethylen as used for the bags does not retain any of the VOCs (memory effects) and thus my make measurements inaccuarate.

This is a fair concern. We have carefully considered all the used materials and chosen those best fit to the purpose. With regard to the mesh bags, the material is the same as in the polyethylene terephthalate (PET) bags that are often used for VOC measurements. Stewart-Jones and Poppy (J Chem Ecol (2006) 32: 845–864) compared using these PET bags to glass containers, and found very good recoveries of compounds in a dynamic sampling setup like ours. Further, the mesh bags used were placed not only in the enclosures in which uptake by soil from the active layer was assessed, but also in the enclosures with only permafrost soil (see Supplementary Fig. 6). This way we could confirm that the uptake observed in the Uptake experiment was due to uptake by soil, not by the mesh bags.

you have used the term 'active layer soil' in some places, I would find a term like 'soil from the active layer' more appropriate, nbut I guess this is a matter of taste.

We have checked the use of terms throughout the manuscript and tried using the most appropriate and clear terms throughout. The term “active layer soil” has been also used by native English speakers, e.g. Kass et al. (Cryosphere (2017) 11: 2943–2955) with a title “In situ nuclear magnetic resonance response of permafrost and *active layer soil* in boreal and tundra ecosystems”.

line 102 omit comma after showed

Done.

REVIEWERS' COMMENTS:

Reviewer #1 (Remarks to the Author):

The work by Kramshøj et al. is novel and seems valuable for the broad scientific audience. All my comments have been well revised by the authors. I do not have any further suggestions and recommend the manuscript to be published.

Reviewer #2 (Remarks to the Author):

The authors have significantly improved this manuscript in response to reviewers' comments and reformatting for this journal's format. Notably, they have promoted the table listing volatile peaks measured to Supplementary Table 1, moved the essential table of soil properties into the main text, and introduced a new Figure 2 that addresses some of the factors correlated with VOC variability in different permafrost samples. They have also clarified interpretation of the results in several parts of the main text. As a result, this work should be more accessible to the permafrost research community.

I think the authors' argument against abiotic formation of VOCs at low temperatures is reasonable given the short periods of analysis. Sterilization experiment controls using autoclaved soil are tricky, as the high heat can change sorption properties and perhaps alter the availability of catalytic mineral surfaces in the sample in addition to inactivating microbes. But that is not a problem unique to this work.
